# Nuclease-induced stepwise photodropping (NISP) to precisely investigate single-stranded DNA degradation behaviors of exonucleases and endonucleases

**DOI:** 10.1093/nar/gkae822

**Published:** 2024-10-01

**Authors:** Hui-Pin Chiu, Chung-Han Shen, Jan-Kai Wu, Eric Y C Mao, Han-Yi Yen, Yuan-Pin Chang, Chyuan-Chuan Wu, Hsiu-Fang Fan

**Affiliations:** Institute of Medical Science and Technology, National Sun Yat-sen University, No. 70, Lien-hai Road, Kaohsiung, 804201, Taiwan; Department of Chemistry, National Sun Yat-sen University, No. 70, Lien-hai Road, Kaohsiung, 804201, Taiwan; Institute of Medical Science and Technology, National Sun Yat-sen University, No. 70, Lien-hai Road, Kaohsiung, 804201, Taiwan; Department of Chemistry, National Sun Yat-sen University, No. 70, Lien-hai Road, Kaohsiung, 804201, Taiwan; Institute of Medical Science and Technology, National Sun Yat-sen University, No. 70, Lien-hai Road, Kaohsiung, 804201, Taiwan; Department of Chemistry, National Sun Yat-sen University, No. 70, Lien-hai Road, Kaohsiung, 804201, Taiwan; Department of Biochemistry and Molecular Biology, College of Medicine, National Cheng Kung University, No. 1, University Road, Tainan, 701, Taiwan; Department of Biochemistry and Molecular Biology, College of Medicine, National Cheng Kung University, No. 1, University Road, Tainan, 701, Taiwan; Department of Chemistry, National Sun Yat-sen University, No. 70, Lien-hai Road, Kaohsiung, 804201, Taiwan; Department of Biochemistry and Molecular Biology, College of Medicine, National Cheng Kung University, No. 1, University Road, Tainan, 701, Taiwan; Institute of Medical Science and Technology, National Sun Yat-sen University, No. 70, Lien-hai Road, Kaohsiung, 804201, Taiwan; Department of Chemistry, National Sun Yat-sen University, No. 70, Lien-hai Road, Kaohsiung, 804201, Taiwan

## Abstract

Here, we employed a fluorescence-based single molecule method called nuclease-induced stepwise photodropping (NISP) to measure in real time the DNA degradation mediated by mitochondrial genome maintenance exonuclease 1 (MGME1), a bidirectional single-stranded DNA (ssDNA)-specific exonuclease. The method detects a stepwise decrease in fluorescence signals from Cy3 fluorophores labeled on an immobilized DNA substrate. Using NISP, we successfully determined the DNA degradation rates of 6.3 ± 0.4 and 2.0 ± 0.1 nucleotides (nt) s^–1^ for MGME1 in the 5′-to-3′ and 3′-to-5′ directions, respectively. These results provide direct evidence of the stronger 5′ directionality of MGME1, consistent with its established role in mitochondrial DNA maintenance. Importantly, when we employed NISP to investigate mung bean nuclease, an ss-specific endonuclease, we observed a markedly different NISP pattern, suggesting a distributive cleavage activity of the enzyme. Furthermore, we applied NISP to determine the ssDNA degradation behavior of the double-stranded-specific exonuclease, λ exonuclease. These findings underscore the capability of NISP to accurately and reliably measure the degradation of ssDNA by both exo- and endonucleases. Here, we demonstrate NISP as a powerful tool for investigating the ssDNA degradation behavior of nucleases at the single-molecule level.

## Graphical Abstract

**Graphical Abstract:**
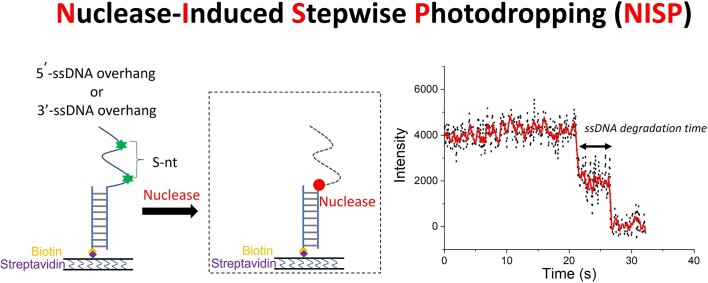

## Introduction

Cells utilize a diverse array of nucleases to regulate and preserve genome function. For instance, single-strand (ss)-specific nucleases play a crucial role in processing 5′ end of newly synthesized DNA strands during DNA replication (1–3), while nucleases capable of performing DNA end restriction and gap generation participate in multiple DNA repair pathways (4,5). Given their significance, single-molecule approaches have been extensively applied to investigate these nucleases and elucidate their intricate mechanisms. For example, the degradation properties of an individual λ exonuclease along a duplex DNA molecule have been studied with fluorescence resonance energy transfer (FRET), optical tweezers (OT) and magnetic tweezers (6–8). Origami-rotor-based imaging and tracking (ORBIT) and tethered particle motion (TPM) have been used to study RecBCD-mediated DNA degradation (9,10). Further, single-molecule DNA curtains have been used to obtain detailed acting mechanisms of several nucleases, including AdnAB, Exo1 and Dna2 (11–13). Moreover, fluorescence enhancement, quenching and FRET have been applied to study flap endonuclease 1 to obtain its substrate-binding and cleavage properties (14–17). These above-mentioned works offer important insights into the working mechanism of the investigated enzyme; however, none of these single-molecule methods can precisely measure the kinetics of ss-specific nucleases without additional enzyme modifications. To acquire such information, researchers often have to label the enzyme with a fluorophore, whether using FRET or DNA curtain (12,5,18). As for bead manipulating systems, such as OT, ORBIT or TPM, bead tethering or surface anchoring of the enzyme is required. These modifications must be done cautiously, as they may introduce unexpected steric hindrances and influence the measured phenomenon (10,19–21). As a result, to study ss-specific nuclease, currently, no suitable single-molecule approaches can be applied without extra modifications of the enzyme.

Mitochondrial genome maintenance exonuclease 1 (MGME1), as suggested by its name, is a mitochondrial nuclease involved in the upkeep of mitochondrial DNA (mtDNA) (22,23). Loss-of-function mutations in the gene encoding MGME1 impair mtDNA integrity and lead to mitochondrial dysfunction in various organs (22,24,25). MGME1 plays roles in both replication and degradation of mtDNA (22,25–28). During mtDNA replication, it collaborates with DNA polymerase γ (Polγ) to process the 5′ end of newly synthesized mtDNA, ensuring accurate DNA end joining to maintain mtDNA integrity (27). During mtDNA degradation, MGME1 is thought to degrade fragmented mtDNA exonucleolytically from the 5′ end in conjunction with mtDNA helicase Twinkle, which unwinds the duplex region (28). *In vitro* studies have characterized MGME1 as a single-stranded DNA (ssDNA)-specific exonuclease capable of degrading ssDNA from both 5′ and 3′ ends (22,23,27,29,30). The characterization has primarily relied on gel electrophoresis-based analysis of cleavage products from bulk reactions, offering an overall view of MGME1-derived DNA cleavage pattern (22,23,29) and some kinetic insights indicating MGME1’s preference for 5′ phosphate, characteristic of a 5′ exonuclease (30). However, the bidirectional nature of MGME1 has complicated and limited the kinetic analysis of the enzyme activity using gel imaging.

Here, we have developed a simple method to investigate ss-specific nuclease-mediated DNA degradation at the single-molecule level without modifying the nuclease. By employing ssDNA labeled with two Cy3 fluorophores, we observed a two-step decrease in fluorescence intensity (photodropping) resulting from the nuclease-mediated sequential removal of the fluorophores. We have named this assay ‘nuclease-induced stepwise photodropping’ (NISP). By using this method, we successfully determined the degradation rate of MGME1 in both the 5′-to-3′ and 3′-to-5′ directions. We found that MGME1 degrades ssDNA from the 5′ end approximately 3.1 times faster, resulting in an overall degradation efficiency about 1.9 times higher than when degrading ssDNA from the 3′ end. These results demonstrate that MGME1 acts as a bidirectional exonuclease with asymmetric polarity. To further explore the application of NISP, an ss-specific DNA/RNA endonuclease, mung bean nuclease (MBN), was also investigated by the method. MBN induced NISP signal in a concentration-dependent manner when incubating with either 5′- or 3′-ssDNA overhangs, indicating a distributive cleavage property of the endonuclease. Moreover, NISP was also adopted to investigate the well-studied double-strand (ds)-specific DNA exonucleases, λ exonuclease, and the ssDNA degradation rate can be precisely determined. This report thus demonstrates that NISP is an efficient and convenient method providing precise measurements of nuclease-mediated ssDNA degradation behavior at the single-molecule level.

## Materials and methods

### Proteins

The recombinant human MGME1 was prepared according to the previous study (31). In brief, the *C20orf72* gene (base pairs 61–1032, encoding residues 21–344, which lacks the N-terminal mitochondrial-targeting sequence) was cloned into pSol vector (Lucigen) to generate the pSol-His_8_-SUMO-MGME1 plasmid. The plasmid was used as the template to introduce K253A and H180A (KH) substitutions by site-directed mutagenesis. *Escherichia coli* BL21 (DE3) pLysS cells harboring the recombinant vectors either expressing wild-type MGME1 or MGME1-KH were grown as liquid culture in the presence of 50 μg ml^−1^ kanamycin and 30 μg ml^−1^ chloramphenicol until OD_600_ reached 0.8–1.0. Protein expression was induced by adding 0.005% l-rhamnose and carried out at 20°C for 16 h. Cells were then harvested and lysed in lysis buffer [50 mM sodium phosphate, pH 8.0, 500 mM NaCl, 10% (v/v) glycerol, 1% (v/v) Tween 20, 0.5 mM phenylmethylsulfonyl fluoride (PMSF), 10 mM imidazole and 5 mM β-mercaptoethanol (β-ME)]. Lysate was cleaned by centrifugation, filtered with 0.45 μm filters and loaded onto a 5-ml HisTrap FF column (Cytiva) pre-equilibrated with wash buffer (lysis buffer containing no Tween 20 and PMSF). After the washing, bound protein was eluted from the column with an elution buffer (wash buffer containing 200 mM imidazole) and treated with TEV protease in a buffer containing 20 mM Tris–HCl (pH 8.0), 200 mM NaCl and 1 mM dithiothreitol (DTT) at 4°C. The sample was further purified by the 5-ml HisTrap FF column to remove the remaining tagged protein and the His6-tagged TEV protease. Tag-free MGME1 was collected, concentrated with 30-kDa cutoff Amicon Ultra Centrifugal Filter Units (Merck) and applied to the HiLoad 16/600 Superdex 200 pg column (Cytiva) pre-equilibrated with buffer containing 20 mM Tris–HCl (pH 8.0), 200 mM NaCl, 5 mM β-ME and 1 mM ethylenediaminetetraacetic acid. The eluted protein was pooled and concentrated. Aliquots of the purified proteins were quickly frozen with liquid N_2_ and stored at −80°C for further use. Protein purity was checked with sodium dodecyl sulfate–polyacrylamide gel electrophoresis. MBN (M0250L) and λ exonuclease (M0262L) were purchased from New England Biolabs.

### Single-molecule measurement acquisition

The reaction chambers were prepared according to the previous paper (32,33). Reaction chambers were coated with streptavidin by incubation with 20 μg/ml streptavidin solution for 10 min at room temperature. Reaction buffer [10 mM HEPES (pH 7.5), 150 mM NaCl, 2.5mM MgCl_2_ for MGME1; 20 mM MES (pH 6.0), 30 mM NaCl, 1 mM ZnCl_2_ for MBN; 67 mM glycine–KOH (pH 9.4), 2.5 mM MgCl_2_ and 50 μg/ml bovine serum albumin (BSA) for λ exonuclease] was flowed to wash out excess streptavidin. Fifty picomolar of biotinylated DNA molecules were flowed in and incubated for 10 min (the sequences and labeling positions of DNA molecules are listed in Supplementary Table S1). DNA substrates used in single-molecule measurement were purchased from MDBio, Taiwan. Unbound DNA molecules were removed with an extensive amount of imaging buffer containing 2 mM Trolox (Sigma–Aldrich), 0.3 mg/ml glucose oxidase (Sigma–Aldrich), 4 mg/ml glucose (Sigma–Aldrich), 0.015 mg/ml catalase (Sigma–Aldrich), 1 mM DTT, 0.1 mg/ml BSA and specific reaction buffer before imaging. NISP and FRET experiments were carried out with an objective-type total internal reflection fluorescence microscope (TIRFM, Nikon eclipse Ti equipped with Di01-R405/488/532/635 and FF01-446/510/581/703-25) and 532 nm laser (DPBL-9020F) as excitation light source. The fluorescence signals of Cy3 were collected by EMCCD (Evolve 512, Photometrics) at 100 ms resolution with a recording window of 100 s for each experiment using self-written software in Labview 2009. In NISP and FRET experiments, we set the laser power to 7 mW to minimize photobleaching events and attain a favorable signal-to-noise ratio according to equation SNR =  (*S*− *B*)/*σ*, where *S* is the average signal intensity, and *B* and *σ* are the average and standard deviation of background intensity, respectively. For NISP experiments, a specific amount of nuclease (MGME1, MBN and λ exonuclease) was prepared in an imaging buffer and flowed into a reaction chamber immobilized with Cy3-labeled DNA molecules to initiate the nuclease-mediated ssDNA degradation process. The fluorescence intensity analysis was calculated with software written in IDL (smFRET package (34)). Only molecules with intensities corresponding to twice the Cy3 intensity will be selected for analysis. In each experiment, >150 molecules will be observed, and a certain percentage, ∼25%, of these molecules will meet that intensity criterion. The stepwise fluorescence photodropping time traces of each Cy3-labeled DNA molecule meeting the criteria were analyzed using a MATLAB program [smFRET package (34)] and plotted using Origin 2023. The distribution of photodropping times obtained without nuclease was fitted with a quartic polynomial function that served as a spontaneous photobleaching background. Later, the distribution of photodropping times obtained in the presence of nuclease was fitted with a Gaussian function plus a quartic polynomial function served as a spontaneous photobleaching background to obtain degradation time.

For FRET experiments, a specific amount of nuclease (MGME1) was prepared in an imaging buffer and flowed into a reaction chamber immobilized with Cy3–Cy5 pair-labeled DNA molecules to initiate the reaction. The fluorescence signals of both Cy3 and Cy5 were collected by EMCCD (Evolve 512, Photometrics) at 100 ms resolution using self-written software in Labview 2009. The experimental data were analyzed with a mapping software written in IDL and further sorted to calculate the FRET efficiency of each interested DNA molecule using the Matlab program. The change in FRET efficiency can signal the degradation/unwinding processes.

## Results

### Using NISP to investigate MGME1-mediated ssDNA degradation

**Figure 1.**
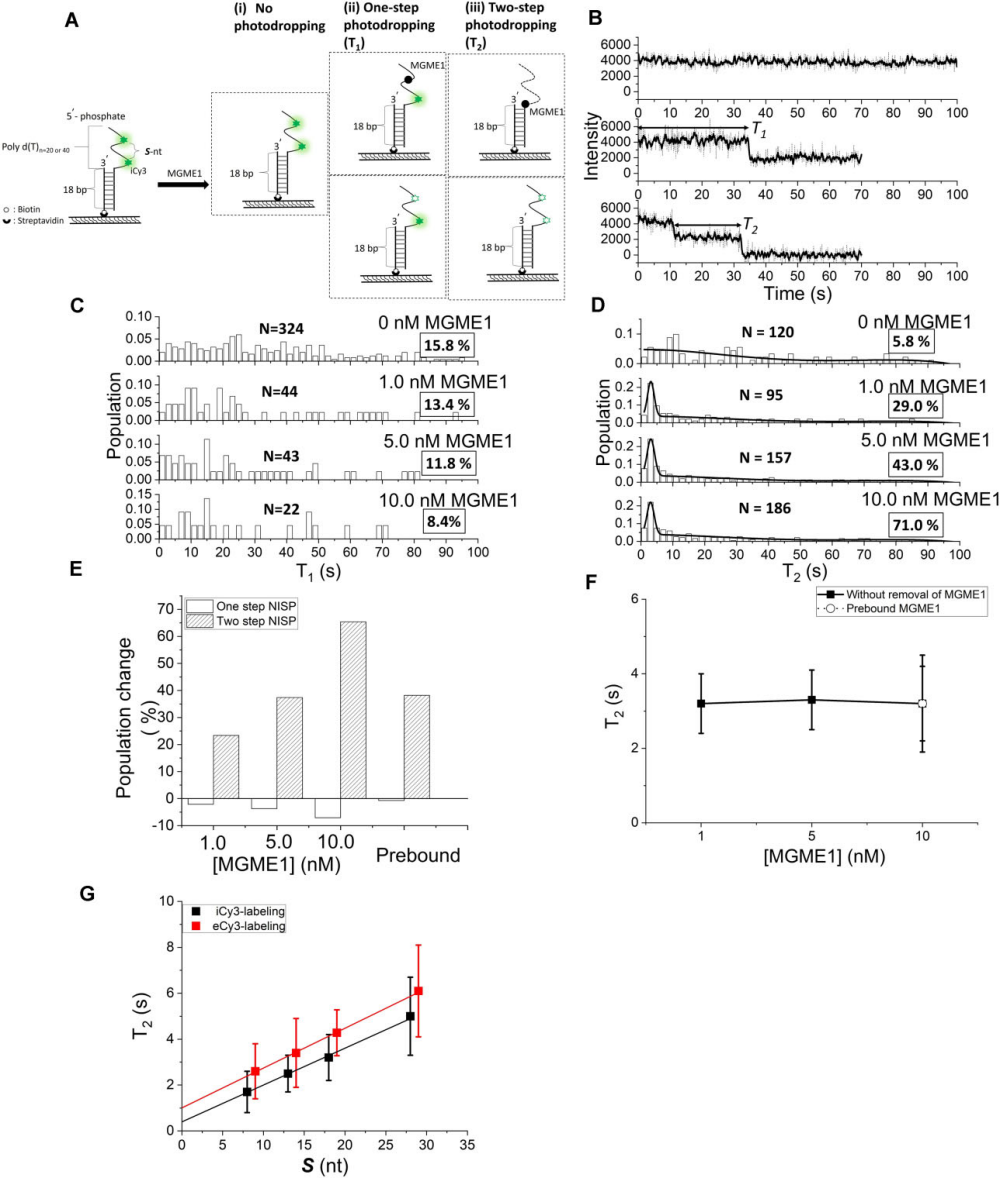
Using NISP to investigate MGME1-mediated 5′-to-3′ ssDNA degradation. (**A**) Schematic of NISP investigating ssDNA degradation by exonuclease. A DNA substrate with a 5′-phosphorylated poly(dT)40 ssDNA overhang is anchored on a PEGylating slide. The ssDNA overhang region is labeled with two internal Cy3 (iCy3) fluorophores in various interval (Supplementary Table S1). ***S*** represents the number of nucleotides with scissile phosphodiester bonds between two Cy3 fluorophores. After injection of exonuclease, ssDNA degradation can be detected by the stepwise photodropping of Cy3 signals. Green asterisks represent iCy3 fluorophores and black circles represent MGME1 nucleases. (**B**) The typical NISP time traces illustrate the behaviors described in panel (A), obtained in the presence of 1 nM MGME1. The dwell times in the one-step NISP time trace (*T*_1_) and two-step NISP time trace (*T*_2_) are marked by double-arrowhead lines. Histograms of *T*_1_ (**C**) and *T*_2_ (**D**) of MGME1 in degrading the 5′-overhang DNA. For panels (C) and (D), *N* indicates the event number, and occurrence probabilities are enclosed by boxes. Solid lines in panel (D) indicate either the nonlinear polynomial fitting for *T*_2_ distribution (in the absence of MGME1) or the nonlinear polynomial plus Gaussian fitting for *T*_2_ distribution (in the presence of MGME1). (**E**) The plots of the population change in the one-step and two-step NISP methods at different concentrations of MGME1, compared to the control scenario without MGME1. (**F**) The plots of averaged *T*_2_ acquired under different concentrations of MGME1 (solid squares; with no extra buffer wash). Open circles represent the experimental data acquired in the Mg^2+^-triggered condition (free MGME1 was removed by buffer wash before triggering the nuclease reaction). (**G**) The plot of averaged *T*_2_, acquired for MGME1 acting on DNA substrates with varying spaces between di-iCy3 fluorophores (black squares) and di-eCy3 fluorophores (red squares), shows a linear fit (*R*^2^= 1.00).

To assay the 5′-exonuclease activity of MGME1, we designed a substrate of duplex DNA carrying a 5′-phosphorylated poly-thymidine (dT) ssDNA overhang. The overhang region was labeled with two Cy3 fluorophores (di-Cy3) separated by a specific interval (Figure 1A and Supplementary Table S1). The substrate is immobilized on a surface, and changes in Cy3 fluorescence intensity are monitored over time in a single-molecule fashion. Within a 100-s acquisition window, the recorded changes in Cy3 fluorescence intensity can be categorized into three scenarios (Figure 1A): (i) no detectable photodropping, indicating no response to the addition of enzyme; (ii) one-step photodropping caused by either enzyme-mediated removal of one Cy3 fluorophore or spontaneous photobleaching; and (iii) two-step photodropping caused by either an enzyme-mediated sequential removal of two Cy3 fluorophores sequentially or spontaneous photobleaching—the latter two scenarios, hereafter, we named one-step NISP and two-step NISP, respectively. The dwell time in the one-step and two-step NISP time trace is, respectively, named *T*_1_ and *T*_2_. *T*_2_ specifically measures the time required for MGME1 to cleave native scissile phosphodiester bonds and an additional bond associated with a Cy3-labeled nucleotide (Supplementary Figure S1). The following assumptions were made. First, the time required for MGME1 to cleave each individual native scissile phosphodiester bond, *T*_2,MGME1_, is constant, irrespective of substrate length variation. Second, the time required for MGME1 to cleave the phosphodiester bond associated with a Cy3-labeled nucleotide, *T*_2,Cy3_, remains constant across different substrate lengths. Third, the observed dwell time *T*_2_ in two-step NISP on di-Cy3 labeled overhang ssDNA substrates correlates with the sum of *T*_2,MGME1_× ***S*** + *T*_2,Cy3_. Here, ***S*** represents the number of nucleotides with scissile phosphodiester bonds between the two Cy3 fluorophores (Figure 1A and Supplementary Table S1). Therefore, by varying the interval length between the two Cy3 fluorophores on the NISP substrates, we aimed to elucidate the kinetics of MGME1-mediated ssDNA degradation.

Previous reports indicate that the position of Cy3 labeling on DNA strands can influence photostability in single molecule detection (35). For this study, DNA probes labeled with either internal Cy3 (in phosphate backbone; termed iCy3) or external Cy3 (on thymine base; termed eCy3) were prepared (Supplementary Figure S1) for NISP. To assess the impact of these labelings on MGME1’s activity, we performed gel electrophoresis analysis of MGME1-derived DNA cleavage product with DNA substrates labeled with iCy3 and eCy3 (Supplementary Figure S2 and Supplementary Text). While iCy3 made minimal effect on the degradation of 5′-overhang substrates by MGME1, eCy3 introduced a more pronounced hindrance (Supplementary Figure S2). Therefore, in subsequent studies, iCy3-labeled DNA was used to set up the NISP assay to initially investigate MGME1-mediated ssDNA degradation. Given that fluorophore can undergo spontaneous photobleaching [the lower panels in the scenarios (ii) and (iii) in Figure 1A], which highly depends on the illumination intensity and experimental environment (e.g. pH and the fluorophore labeling position) (36), we have optimized our system and found an optimal illumination intensity that yielded a good signal-to-noise ratio with minor spontaneous photobleaching probability (Supplementary Figure S3). To further subtract the spontaneous photobleaching from the enzyme-mediated events, we monitored the photodropping of the substrate without adding enzymes, yielding the occurrence probabilities of 78.4%, 15.8% and 5.8% for the above-mentioned scenarios (i), (ii) and (iii), respectively (Table 1 and Supplementary Figure S4). The measurement served as the baseline to be subtracted from the enzyme-added conditions (Figure 1B–D) to obtain the change in one-step and two-step NISP populations (Figure 1E). We found that adding 1 nM MGME1 to the system did not change the one-step NISP population significantly (Figure 1C and E), indicating that the observed *T*_1_ purely represented spontaneous photobleaching of one Cy3 fluorophore. In contrast, the occurrence probability of two-step NISP increased vastly by 23.2% in the presence of 1 nM MGME1 (Figure 1E and Table 1). Further, in the presence of 10 nM MGME1, the growth in the two-step NISP population reached 65.2%, accompanied by a corresponding decrease of 7.4% in the one-step NISP population. These observations indicate that MGME1 mainly causes two-step NISP events, presumably resulting from the gradual 5′-to-3′ degradation of the 5′-ssDNA overhangs, as we expected. To further confirm whether the NISP events fully represent the activity of MGME1, we replaced the catalytic ion (Mg^2+^) in the reaction buffer with Ca^2+^, which acts as an inhibitory ion for MGME1 (29). In this condition, the occurrence probability of the three scenarios was similar to that of the control without adding enzymes, confirming that the NISP signal was strictly dependent on the nuclease's activity (Supplementary Figure S5A–C). A catalytically inactive MGME1 carrying H180A and K253A substitutions, herein termed MGME1-KH (Supplementary Figure S6), was also assayed by NISP (Supplementary Figure S5D–F). These two residues are known to participate in MGME1 catalysis (22,23,26,29). As expected, no NISP signal was observed for MGME1-KH (Supplementary Figure S5D–F). Together, these results demonstrate that NISP can be used to investigate the ssDNA degradation behavior of exonucleases at the single-molecule resolution.

**Table 1.** The probabilities distribution of the three scenarios in NISP induced by MGME1

			Probability (%)			
Proceeding direction	[MGME1] (nM)	***S*** (nt)	No photodropping	One-step NISP	Two-step NISP	*T*_2_ (s)
5′-to-3′	0	18	78.4 (1611/2055)	15.8 (324/2055)	5.8 (120/2055)	N.D.^a^
	1.0		57.6 (189/328)	13.4 (44/328)	29.0 (95/328)	3.2 ± 0.8
	5.0		45.2 (165/365)	11.8 (43/365)	43.0 (157/365)	3.3 ± 0.8
	10.0		20.6 (54/262)	8.4 (22/262)	71.0 (186/262)	3.2 ± 1.0
	Prebound		40.9 (106/259)	15.1 (39/259)	44.0 (114/259)	3.2 ± 1.3
3′-to-5′	0	18	79.9 (1348/1688)	14.3 (242/1688)	5.8 (98/1688)	N.D.^a^
	1.0		65.8 (285/433)	15.9 (69/433)	18.3 (79/433)	10.6 ± 1.4
	5.0		48.4 (132/273)	15.7 (43/273)	35.9 (98/273)	10.5 ± 1.9
	10.0		31.7 (58/183)	15.3 (28/183)	53.0 (97/183)	10.4 ± 1.9
	Prebound		56.7 (157/277)	17.3 (48/277)	26.0 (72/277)	10.5 ± 1.9

^a^N.A., not assigned due to the low NISP probabilities.

Within these data, we ought to isolate *T*_2_ values that purely represent MGME1-mediated degradation, excluding the effects of spontaneous photobleaching. First, the obtained *T*_2_ values were pooled to create *T*_2_ histograms with different bin sizes and fitted with Gaussian distributions to determine which bin size can yield a consistent and reliable average degradation time (*T*_2,av_) value (Supplementary Figure S7). Then, the *T*_2_ histograms were built with a specific bin size in order to proceed following analysis. Second, the *T*_2_ histograms in various MGME1 concentrations were fitted with a Gaussian distribution to obtain *T*_2,av,Gaussian_ of 3.1 ± 0.8 s (Supplementary Figure S8A). Third, to subtract spontaneous photobleaching, the *T*_2_ histograms in the presence of MGME1 were fitted with a Gaussian function plus a quartic polynomial function that served as spontaneous photobleaching background, yielding a *T*_2,av,Gaussian+polynomial_ of 3.2 ± 0.6 s (Supplementary Figure S8B), which exhibits an ∼3.1% difference from that obtained without background subtraction. Hereafter, we employed this background-subtracted methodology to derive *T*_2_ values that accurately represent MGME1-mediated degradation, thus effectively excluding the impact of spontaneous photobleaching. The derived *T*_2_ value correlates with the sum of *T*_2,MGME1_× ***S*** + *T*_2,Cy3_, where ***S*** denotes the number of nucleotides with scissile phosphodiester bonds between the two Cy3 fluorophores (Figure 1A and Supplementary Table S1). With DNA substrates named 5′-to-3′ di-iCy3-18nt (Supplementary Table S1), we determined *T*_2_ induced by different concentrations of MGME1, obtaining the values of 3.2 ± 0.8, 3.3 ± 0.8 and 3.2 ± 1.0 s for the conditions of adding 1.0, 5.0 and 10.0 nM MGME1, respectively (Table 1). As shown by the result, the background-subtracted *T*_2_ did not change upon varying MGME1 concentration (Figure 1F), indicating that we have been observing a single-turnover event. The result also suggests that MGME1 can degrade the 5′-ssDNA overhang processively, as exemplified by the single-molecular studies on other enzymes (6,37). To further confirm the processibility of MGME1-mediated ssDNA cleavage, 10 nM MGME1 was preincubated with the DNA substrate in the absence of Mg^2+^ for 10 min. Subsequently, free MGME1 was removed from the system by flowing out of the reaction chamber with extra buffer, followed by flowing in Mg^2+^ to trigger the cleavage process. The resultant *T*_2_ was 3.2 ± 1.3 s (Figure 1F, open circles), which is comparable to the result obtained without removing the free MGME1 prior to triggering the cleavage reaction (Figure 1F, solid squares), supporting that MGME1 degrades the 5′-ssDNA overhang processively.

As mentioned, we assume that the *T*_2_ shall be composed of *T*_2,MGME1_ and *T*_2,Cy3_, where *T*_2,MGME1_ represents the time required for the exonuclease to process one nucleotide with the scissile phosphodiester bond, whereas *T*_2,Cy3_ is the time for removing the Cy3-conjugated nucleotide that should be a constant parameter regardless of varying ***S***. In the case of iCy3-labeled DNA substrates, we named the parameter *T*_2,iCy3_. To verify this assumption, a series of 5′-overhang DNA substrates with different ***S*** were used for monitoring *T*_2_ induced by MGME1 (Supplementary Figure S9 and Table 2), and the value was plotted as a function of ***S***. As we expected, this plot displayed a linear relationship between *T*_2_ and ***S*** (black squares in Figure 1G), where the *y*-intercept was 0.40 ± 0.10 s, which represents *T*_2,iCy3_. The derived slope for the linear fit was 0.16 ± 0.01 s nt^–1^, representing an average ssDNA degradation rate of 6.3 ± 0.4 nt s^–1^ by MGME1 as it works in the 5′-to-3′ direction.

**Table 2.** The probabilities distribution of the three scenarios in NISP determined ssDNA degradation rate of MGME1 on DNA substrates labeled with either iCy3 or eCy3 fluorophores separated by different number of nucleotides with scissile phosphodiester bonds between the two Cy3 fluorophores (***S***)

				Probability (%)			
Proceeding direction	Labeling	***S*** (nt)	[MGME1] (nM)	No photodropping	One-step NISP	Two-step NISP	T_2_ (s)
5′-to-3′	iCy3	8	10	13.9 (31/223)	9.9 (22/223)	76.2 (170/223)	1.7 ± 0.9
		13		17.4 (34/196)	11.7 (23/196)	70.9 (139/196)	2.5 ± 0.8
		18		20.6 (54/262)	8.4 (22/262)	71.0 (186/262)	3.2 ± 1.0
		28		31.3 (71/227)	16.3 (37/227)	52.4 (119/227)	5.0 ± 1.7
	eCy3	9		17.3 (19/110)	12.7 (14/110)	70.0 (77/110)	2.6 ± 1.2
		14		20.4 (22/108)	12.0 (13/108)	67.6 (73/108)	3.4 ± 1.5
		19		22.8 (113/496)	10.9 (54/496)	66.3 (329/496)	4.3 ± 1.0
		29		28.7 (102/355)	20.3 (72/355)	51.0 (181/355)	6.1 ± 2.0
3′-to-5′	iCy3	8		24.3 (46/189)	14.3 (27/189)	61.4 (116/189)	5.0 ± 1.1
		13		25.7 (54/210)	17.6 (37/210)	56.7 (119/210)	7.6 ± 1.9
		18		31.7 (58/183)	15.3 (28/183)	53.0 (97/183)	10.4 ± 1.9
		28		34.2 (67/196)	19.4 (38/196)	46.4 91/196)	14.9 ± 2.0
	eCy3	9		33.9 (60/177)	17.0 (30/177)	49.1 (97/177)	6.0 ± 2.4
		14		37.2 (68/183)	17.5 (32/183)	45.3 (83/183)	8.5 ± 1.5
		19		30.4 (136/447)	22.6 (101/447)	47.0 (210/447)	11.7 ± 1.6
		29		44.1 (82/186)	22.6 (42/186)	33.3 (62/134)	16.2 ± 1.7

This result indicates that MGME1 required a longer time to process the iCy3-conjugated nucleotide (0.40 ± 0.10 s) than processing a normal nucleotide (0.16 ± 0.01 s). Indeed, a notable accumulation of cleavage intermediates before the iCy3 site was observed when we inspected the MGME1-derived cleavage pattern from a DNA substrate carrying a single iCy3 in the middle of the 14-nt 5′ overhang (Supplementary Figure S2A and B). To further evaluate the effect of eCy3 modification on the observed *T*_2_, the same experimental scheme was conducted with a different set of DNA substrates carrying eCy3 fluorophores (Supplementary Figure S10 and Table 2), and the time required for removing the fluorophore was named *T*_2,eCy3_. Strikingly, by plotting *T*_2_ as a function of ***S*** (red squares in Figure 1G), we obtained a linear relationship between *T*_2_ and ***S*** with the derived slope of 0.17 ± 0.01 s nt^–1^, similar to the value acquired from the iCy3-labeled substrates. The *y*-intercept of the eCy3 substrate-derived plot was 1.00 ± 0.24 s, indicating that MGME1 requires even more time to process the eCy3-conjugated nucleotide than the iCy3-conjugated nucleotide. This result is well in line with our observation from the DNA cleavage pattern derived from the eCy3-labeled DNA overhang (Supplementary Figure S2A, B and E), where more accumulation of the cleavage intermediates was found. Collectively, these results demonstrate that the specific influences of each fluorophore conjugation can be independently subtracted from *T*_2_ to isolate the exact time for the exonuclease to process native nucleotides. Accordingly, the 5′-to-3′ ssDNA degradation rate of MGME1 was determined from the inverse of the slope in the linear regression, yielding a degradation rate of 6.3 ± 0.4 nt s^–1^ (Figure 1F).

### MGME1 is an ssDNA-specific enzyme

**Figure 2.**
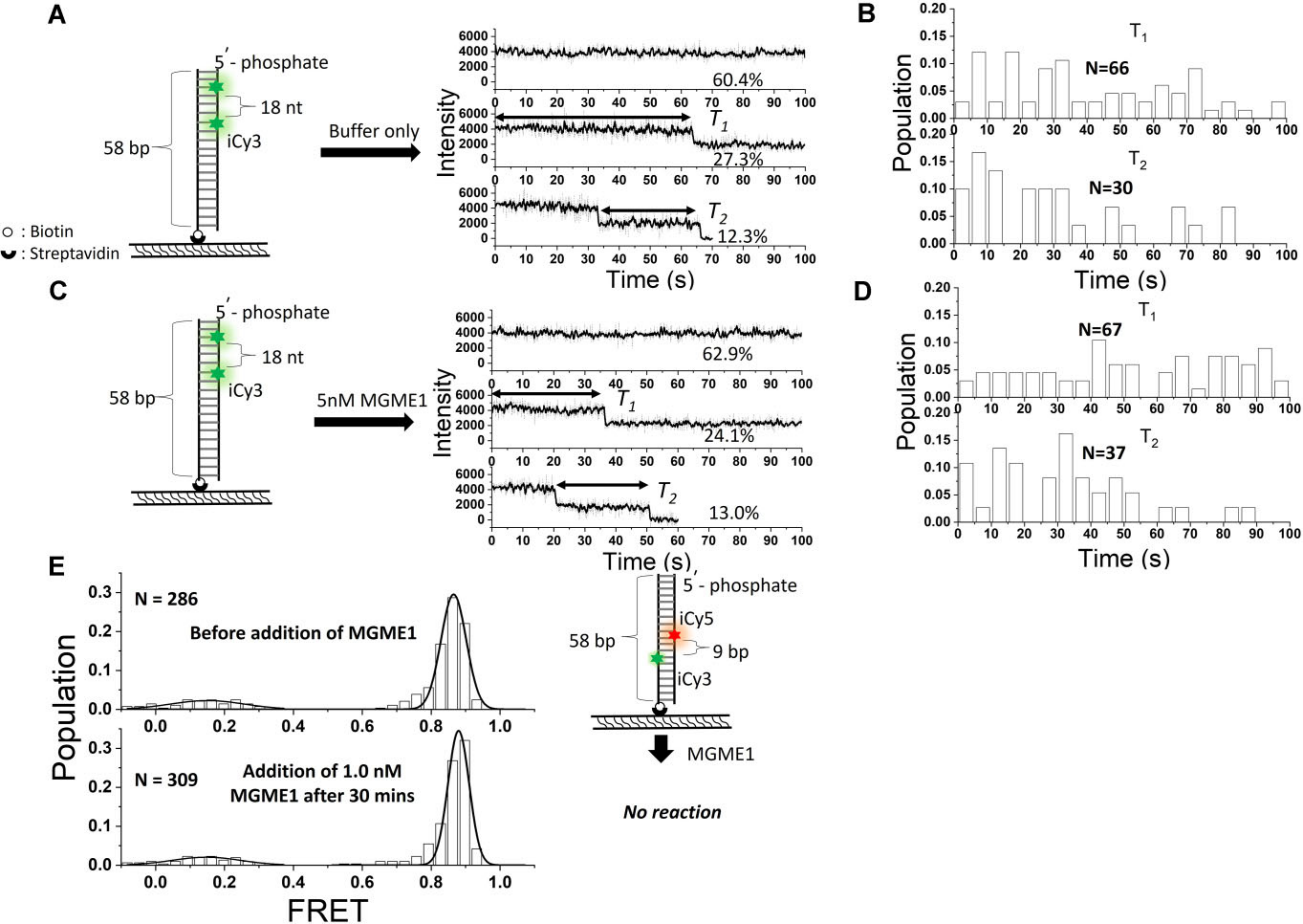
MGME1 cannot degrade blunt-end dsDNA. (**A**–**D**) Using NISP to investigate MEMG1’s activity on dsDNA. A DNA named 5′→3′ di-iCy3-18nt dsDNA was used, and the number of nucleotides with scissile phosphodiester bonds between two Cy3 fluorophores is 18 nt (Supplementary Table S1). NISP time traces in the absence (**A**) and presence of 5 nM MGME1 (**C**) are shown. *T*_1_ and *T*_2_ are marked with double arrowhead lines. Panels (B) and (D) show the histograms of *T*_1_ and *T*_2_ in the absence and presence of 5 nM MGME1, respectively. *N* indicates the event number. (**E**) Using FRET to investigate MGME1’s activity on dsDNA. Histograms of the FRET efficiency before (upper) and after adding 5.0 nM MGME1 (lower) are shown. *N* indicates the event number. Schematics of the DNA substrates used in the FRET assay are shown in the right panels. Green asterisks represent iCy3 fluorophores and red asterisks represent iCy5 fluorophores.

Structural analysis of MGME1 revealed a ring-shaped architecture with a selective hole that allows only ssDNA to thread through the protein and access the catalytic center of the nuclease (29,31). Due to this steric hindrance, MGME1 would obligatorily stop degradation around the single-stranded–double-stranded (ss–ds) DNA junction (Supplementary Figures S2 and S11) and possess very limited activity toward duplex region (22,23,27,30,31). To further verify this property at single-molecule resolution, a DNA substrate named 5′→3′ di-iCy3-18nt dsDNA was used (Figure 2A and Supplementary Table S1) for NISP assay. After adding the reaction solution without or with 5.0 nM MGME1, the representative time traces, occurrence probabilities and histograms of *T*_1_ and *T*_2_ were measured (Figure 2). The occurrence probability of two-step NISP events in the presence of 5.0 nM MGME1 was 13.0% (Figure 2C), similar to that of MGME1-free condition (12.3%, Figure 2A). No significant differences in *T*_2_ were shown in response to adding enzymes (Figure 2B and D). In contrast to the substantial increase in the two-step NISP signal when incubating 5.0 nM MGME1 with the 5′-overhang DNA (Figure 1E), this observation indicates that MGME1 exhibits very limited ability to process blunt-end duplex DNA. Furthermore, a FRET assay was conducted with a 58-bp, blunt-end dsDNA labeled with an iCy3–internal Cy5 (iCy5) pair separated by 9 bp (Supplementary Table S1, DNA substrate named FRET-dT_9_ dsDNA). The probe exhibits a high FRET efficiency of 0.85 ± 0.02 (Figure 2E, upper panel). The unwinding of the duplex DNA or digestion of the labeled strand will result in the loss of iCy5 fluorophore and decrease the high FRET population. After adding 1.0 nM MGME1, the histogram of FRET efficiency showed no detectable difference compared to that of enzyme-free condition (Figure 2E, lower panel). Taking together, by using two different types of single-molecule methods, here we confirm that MGME1 is an ss-specific nuclease and has no activity toward blunt-end dsDNA.

### MGME1 exhibits a weaker ability to degrade ssDNA in the 3′-to-5′ direction

**Figure 3.**
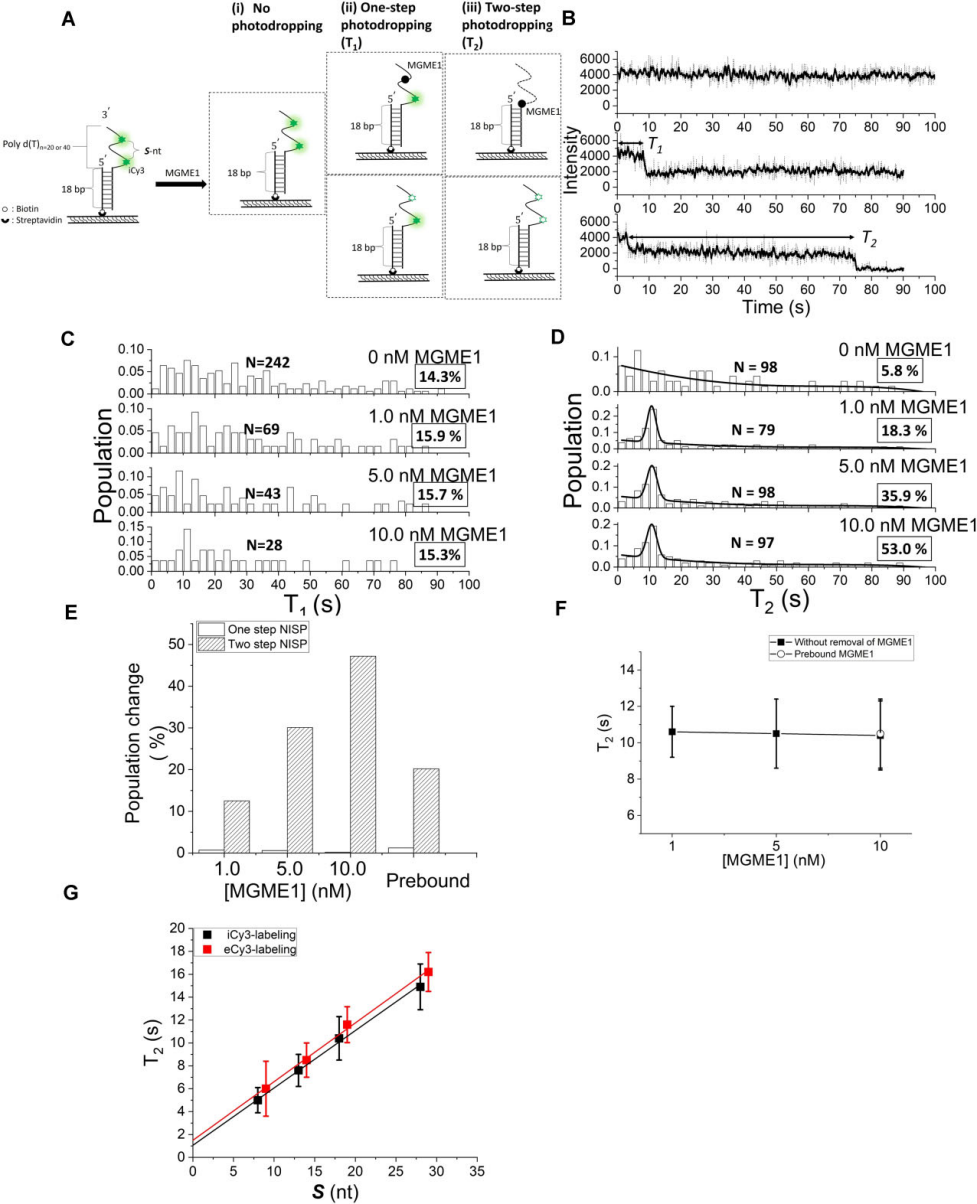
Using NISP to investigate MGME1-mediated 3′-to-5′ ssDNA degradation. (**A**) Schematic of the 3′-poly (dT)_40_ overhang DNA substrate used in NISP. The ssDNA overhang region is labeled with two internal Cy3 (iCy3) fluorophores in various intervals (Supplementary Table S1). ***S*** represents the number of nucleotides with scissile phosphodiester bonds between two Cy3 fluorophores. Green asterisks represent iCy3 fluorophores and black circles represent MGME1 nucleases. (**B**) The typical NISP time traces obtained from the 3′-overhang DNA substrate in the presence of 1 nM MGME1 as shown in panel (A). *T*_1_ and *T*_2_ are marked with double-arrowhead lines. Histograms of *T*_1_ (**C**) and *T*_2_ (**D**) of MGME1 in degrading the 3′-overhang substrates. *N* indicates the event number, and occurrence probabilities are enclosed by boxes. Solid lines in penal (D) indicate either the nonlinear polynomial fitting for *T*_2_ distribution (in the absence of MGME1) or the nonlinear polynomial plus Gaussian fitting for *T*_2_ distribution (in the presence of MGME1). (**E**) The plots of the population changes in the one-step and two-step NISP methods at different concentrations of MGME1, compared to the control scenario without MGME1. (**F**) The plots of averaged *T*_2_ acquired under different concentrations of MGME1 (solid squares; with no extra buffer wash). Open circles represent experimental data acquired in the Mg^2+^-triggered condition (free MGME1 was removed by buffer wash before triggering the nuclease reaction). **(G)** The plot of averaged *T*_2_, acquired for MGME1 acting on DNA substrates with varying spaces between di-iCy3 fluorophores (black squares) and di-eCy3 fluorophores (red squares), shows a linear fit (*R*^2^= 1.00).

*In vitro*, MGME1 is capable of degrading ssDNA from either end but has a stronger preference for processing ssDNA from the 5′ end (see Supplementary Figures S2 and S11, and Supplementary Text for more descriptions of the data) (22,30). To investigate the unique bidirectionality of MGME1 at single-molecule resolution, DNA substrates carrying 3′-ssDNA overhang were designed for NISP (Figure 3A and Supplementary Table S1). With DNA substrates named 3′-to-5′ di-iCy3-18nt, the spontaneous photobleaching events were measured in the absence of MGME1, giving the occurrence probabilities of 79.9%, 14.3% and 5.8% for no NISP, one-step and two-step NISP, respectively (Table 1). We observed that MGME1 could also induce NISP signal on the 3′-ssDNA overhang substrate (Figure 3), showing that it possessed 3′-exonuclease activity. However, adding 1 nM MGME1 only introduced a 12.5% increase in the two-step NISP signal in degrading the 3′-ssDNA overhang substrates, compared to the 23.2% increase when digesting the 5′-ssDNA overhang substrate (Table 1). Even at higher enzyme concentrations, MGME1 still could not induce the two-step NISP signal from the 3′-ssDNA substrates as efficiently as from the 5′-ssDNA overhang substrates (Figure 3C–E). With DNA substrates named 3′-to-5′ di-iCy3-18nt, we determined background-subtracted *T*_2_ induced by different concentrations of MGME1, obtaining the values of 10.6 ± 1.4, 10.5 ± 1.9 and 10.4 ± 1.9 s for the conditions of adding 1.0, 5.0 and 10.0 nM MGME1, respectively (Figure 3F and Table 1). Therefore, despite being less efficient, we found that MGME1 still digests ssDNA processively in the 3′-to-5′ direction, evidenced by the enzyme concentration-independent *T*_2_. Also, removing the free MGME1 from the system before triggering the cleavage reaction did not change the measured degradation rate (Figure 3F, open circles), again indicating a single-molecular event.

Following the same experimental and data processing procedure (Supplementary Figure S12), we decomposed *T*_2_ into *T*_2,MGME1_ and *T*_2,iCy3_ with the iCy3-labeled substrates varying in ***S***, yielding a linear plot of *T*_2_ against ***S*** with a *y*-intercept of 1.06 ± 0.24 s (Figure 3G, black squares). eCy3-labeled 3′-overhang substrates were also employed for evaluating the influence of eCy3 modification, resulting in a linear relationship of *T*_2_ and ***S*** with the *y*-intercept to be 1.49 ± 0.49 s (Figure 3G, red squares, and Supplementary Figure S13). Importantly, in addition to our observation on the 5′-overhang substrates, we obtained the same derived slopes, ∼0.51 s nt^–1^, from the linear fits of using either iCy3- or eCy3-labeled DNA substrates (Figure 3G). Accordingly, the 3′-to-5′ ssDNA degradation rate of 2.0 ± 0.1 nt s^–1^ was determined by inverting the slope (Figure 3G and Table 2), which is substantially slower than that of MGME1 in digesting ssDNA from the 5′ end.

Taking together, our data suggest that MGME1 also acts as a processive exonuclease in degrading ssDNA from 3′ end but exhibits a significantly slower degrading rate than working in the 5′-to-3′ direction. The *in vitro* characterization thus essentially agrees with the known biological role of MGME1 in mtDNA replication and degradation, in which the enzyme is obligatory to work in the 5′-to-3′ direction (22,23,26,28). Whether the 3′-exonuclease activity has biological function is elusive.

### Application of NISP on ss-specific endonuclease: mung bean nuclease acts as a distributive endonuclease on ssDNA overhang

**Figure 4.**
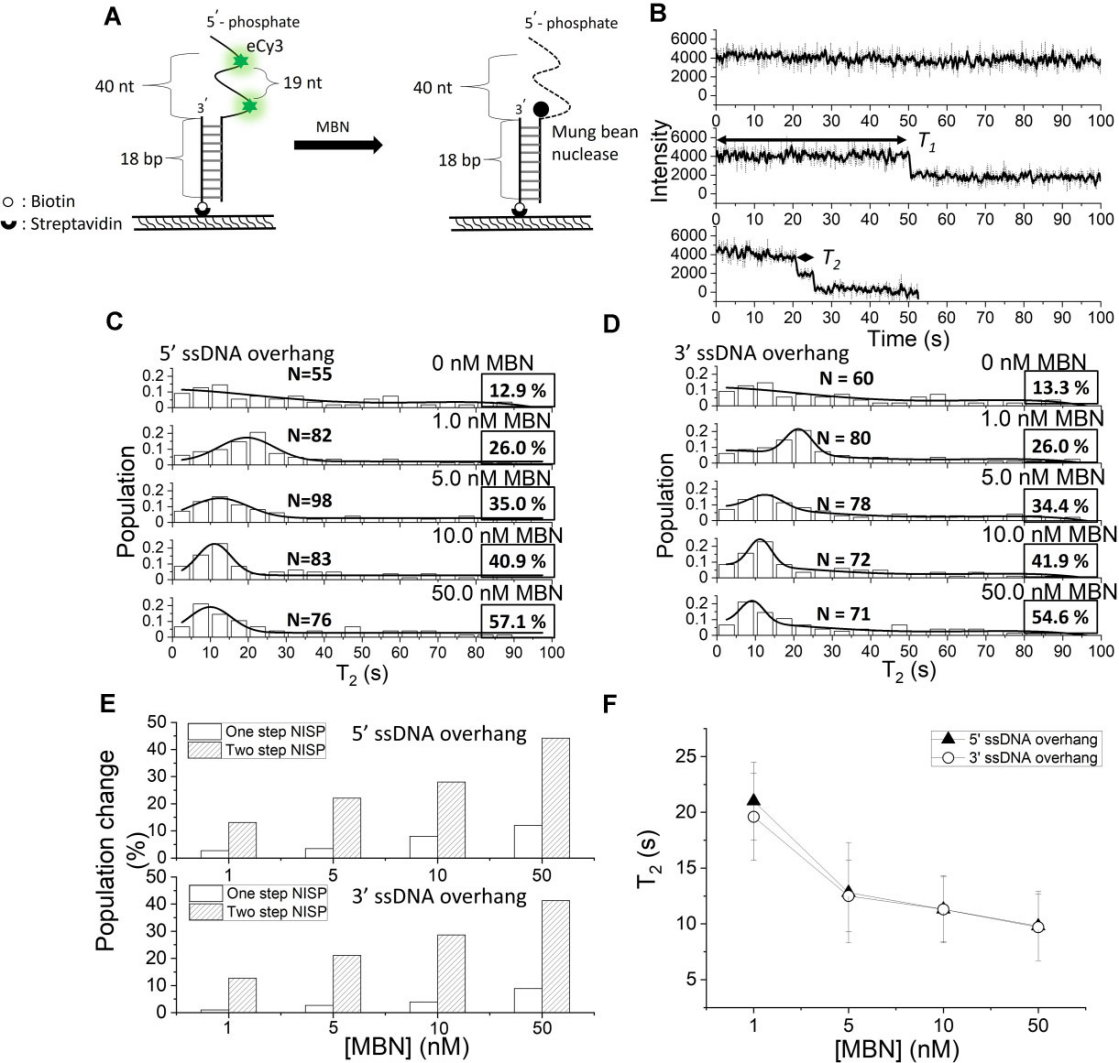
Using NISP to investigate MBN-mediated ssDNA degradation. (**A**) Schematic of the assay with DNA substrate named 5′→3′ di-eCy3-19nt (Supplementary Table S1). The number of nucleotides with scissile phosphodiester bonds between two eCy3 fluorophores is 19 nt. Green asterisks represent eCy3 fluorophores and black circles represent MBN. (**B**) The typical NISP time traces of the used substrate in the presence of 5 nM MBN. *T*_1_ and *T*_2_ are marked with double-arrowhead lines. Histograms of *T*_2_ of MBN in degrading the (**C**) 5′-overhang substrates and (**D**) 3′-overhang substrates. *N* indicates the event number, and occurrence probabilities are enclosed by boxes. The solid lines in panels (C) and (D) indicate either the nonlinear polynomial fitting to *T*_2_ distribution (in the absence of MBN) or the nonlinear polynomial plus Gaussian fitting to *T*_2_ distribution (in the presence of MBN). (**E**) The plots of the population changes in the one-step and two-step NISP methods at different concentrations of MBN along 5′- and 3′-ssDNA overhangs, compared to the control scenario without MBN. (**F**) The plots of averaged *T*_2_ acquired under different concentrations of MBN along 5′- and 3′-ssDNA overhangs.

According to previously reported literature, MBN is a ssDNA/RNA-specific endonuclease (38). To verify the capability of NISP to probe endonuclease behavior, we applied the assay to investigate the catalytic activity of MBN. Examination of the cleavage pattern derived from the enzyme upon the Cy3-labeled probes revealed no significant difference in cleavage efficiency (Supplementary Figure S14 and Supplementary Text), indicating that the Cy3 labeling did not cause substantial interference on MBN-mediated ssDNA degradation. Accordingly, the 5′-ssDNA overhang DNA substrate named 5′→3′ di-eCy3-19nt was used to investigate the activity of MBN (Figure 4A and B, and Supplementary Table S1).

In contrast to the observation of MGME1, through NISP, we found a completely different pattern of the NISP result derived by MBN. First, we found a simultaneous increase in the occurrence probabilities of both one-step and two-step NISP as the concentration of MBN increased (Figure 4C–E). Second, *T*_2_ decreased upon increasing MBN concentration, indicating a non-processive action (Figure 4F and Table 3). The above observation strongly suggests that the enzyme cleaved the substrate in a distributive manner (6,37), which was a typical property of endonuclease. Parallel experiments using the 3′-ssDNA overhang substrates resulted in similar results to those using the 5′- ssDNA overhang substrate (Figure 4D–F and Table 3), confirming that MBN digested ssDNA in a distributive manner. Collectively, by the striking difference between the two types of NISP results derived from exonuclease and endonuclease, our data demonstrated that NISP can be applied to dissect the ssDNA degradation manners of the distinct nuclease activities.

**Table 3.** The probabilities distribution of the three scenarios in NISP induced by MBN

		Probability (%)			
Substrate	[Mung beam nuclease] (nM)	No photodropping	One-step NISP	Two-step NISP	*T*_2_ (s)
5′-ssDNA overhang	0	66.0 (281/426)	21.1 (90/426)	12.9 (55/426)	N.D.^a^
	1.0	50.2 (158/315)	23.8 (75/315)	26.0 (82/315)	21.0 ± 3.5
	5.0	40.4 (113/280)	24.6 (69/280)	35.0 (98/280)	12.8 ± 4.5
	10.0	30.0 (61/203)	29.1 (59/203)	40.9 (83/203)	11.3 ± 3.0
	50.0	9.8 (13/133)	33.1 (44/133)	57.1 (76/133)	9.8 ± 3.1
3′-ssDNA overhang	0	65.6 (296/451)	21.1 (95/451)	13.3 (60/451)	N.D.^a^
	1.0	51.9 (160/308)	22.1 (68/308)	26.0 (80/308)	19.6 ± 3.9
	5.0	41.8 (95/227)	23.8 (54/227)	34.4 (78/227)	12.5 ± 3.2
	10.0	33.1 (57/172)	25.0 (43/172)	41.9 (72/172)	11.3 ± 2.9
	50.0	15.4 (20/130)	30.0 (39/130)	54.6 (71/130)	9.7 ± 3.0

^a^N.A., not assigned due to the low NISP probabilities.

### Application of NISP on dsDNA-specific exonuclease: the ssDNA degradation rate of λ exonuclease can be precisely determined separated from its dsDNA unwinding activity

**Figure 5.**
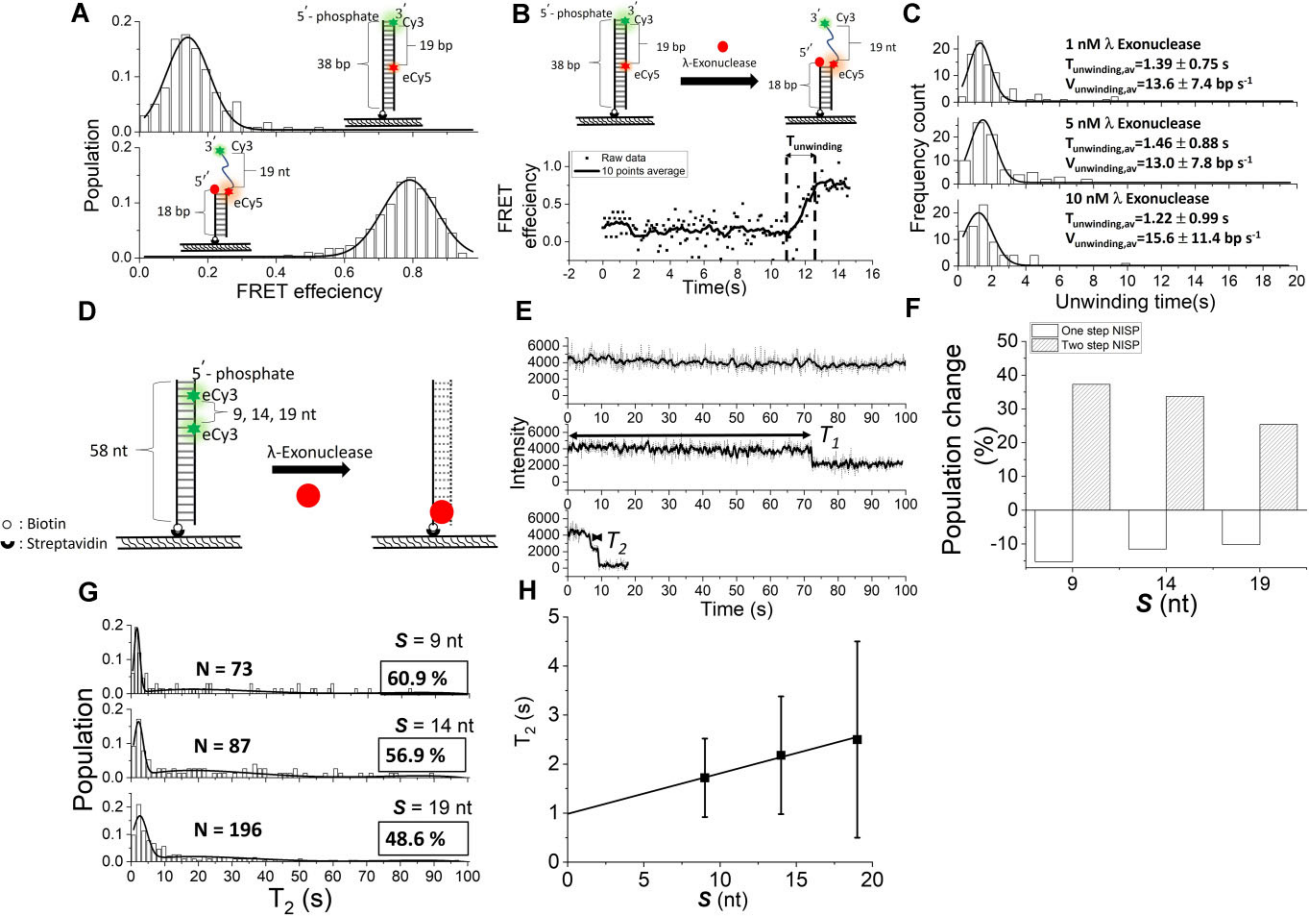
The investigation of λ exonuclease-mediated dsDNA unwinding and ssDNA degradation processes. (A–C) Using FRET to investigate the activity of λ exonuclease. (**A**)FRET histograms of the dsDNA and 3′-overhang DNA used in the assay in the enzyme-free condition. (**B**) The representative FRET efficiency time trace and the schematic to illustrate the λ exonuclease-mediated dsDNA unwinding process. (**C**) The histograms of unwinding times at different concentrations of the exonuclease. (D–H) Using NISP to investigate the activity of λ exonuclease. (**D**) Schematic and (**E**) the typical NISP time traces of the used substrates, respectively. *T*_1_ and *T*_2_ are marked with double-arrowhead lines. (**F**) The population changes in the one-step and two-step NISP methods at different concentrations of λ exonuclease compared to the control scenario without λ exonuclease. (**G**) Histograms of *T*_2_ of λ exonuclease in degrading the eCy3-labeled substrates. The solid lines indicate the nonlinear polynomial plus Gaussian fitting to *T*_2_ distribution obtained from DNA substrates labeled with di-eCy3 spaced differently. **(H)** The plot of averaged *T*_2_, acquired for λ exonuclease acting on DNA substrates with varying spaces between di-eCy3 fluorophores (black suqares), shows a linear fit (*R*^2^= 1.00).

Single-molecule approaches have been widely applied to study the reaction mechanisms of nucleic acid-processing enzymes, including various kinds of nuclease. Interestingly, some of the nucleases, like λ exonuclease, possess both ssDNA degradation and dsDNA unwinding activities, therefore being capable of removing one of the ssDNA strands from dsDNA. However, by using single-molecule methods, so far, only dsDNA unwinding rate or DNA translocation rates have been reported for such nucleases (39,8,6,9,40) FRET assay and NISP assay were adapted here to investigate λ exonuclease-mediated DNA degradation behaviors, dsDNA unwinding rate and ssDNA degradation rate separately. By using FRET assay, a DNA substrate named FRET-19bp dsDNA was used and a low FRET value of 0.14 ± 0.03 was observed here (Figure 5A(i) and Supplementary Table S1). After the addition of 10.0 nM λ exonuclease, the unwinding of dsDNA by λ exonuclease results in a decrease in the time-averaged distance between the Cy3–Cy5 pair and leads to an increase in FRET value to 0.79 ± 0.04 (Figure 5A (ii)). A representative single-molecule FRET time trace exhibited a gradual increase in FRET value from 0.15 ± 0.03 to 0.77 ± 0.03, representing the λ exonuclease-mediated dsDNA unwinding process. The dwell time from minimum FRET to maximum FRET without pause is defined as dsDNA unwinding time, *T*_unwinding_ (Figure 5B), and can be used to calculate the dsDNA unwinding rate, *V*_unwinding_. A series of FRET experiments were conducted in the presence of various concentrations of λ exonuclease from 1 to 10 nM, and the histograms of *T*_unwinding_ were obtained (Figure 5C). A concentration-independent dwell time of ∼1.4 s for λ exonuclease to unwind 19-bp dsDNA was obtained. The dsDNA unwinding rate, the number of base pair unwound (19 bp) divided by the dsDNA unwinding time, of 13.6 ± 9.4 bp s^–1^ was obtained (Figure 5C, Global fitting by Origin), consistent with previously reported values (39,8,6,41).

In order to investigate ssDNA degradation rate of λ exonuclease, the NISP assay was adopted here. To check the interference of Cy3 labeling toward λ exonuclease’s degradation activity, duplex DNA substrates without or with eCy3 or iCy3 labeling were used and investigated in bulk nuclease assay side-by-side here. A delay in λ exonuclease-mediated ssDNA degradation was observed on the DNA labeled with eCy3, indicated by the accumulation of degradation intermediates near the eCy3 labeling site (Supplementary Figure S15 and Supplementary Text). Surprisingly, λ exonuclease was unable to degrade iCy3-labeled DNA substrates, suggesting that the linkage between iCy3 and the nucleotide might interfere with the active site’s interaction with the scissile phosphate and the adjacent nucleotides’ protein–DNA backbone as they approach the active site during processive exonucleolysis. Therefore, 58-bp dsDNA substrates with two eCy3 fluorophores spaced at varying intervals were employed to determine the ssDNA degradation rate of λ exonuclease (Figure 5D and Supplementary Table S1). After adding 5.0 nM λ exonuclease, the degradation of ssDNA by λ exonuclease in 5′-to-3′ direction results in the removal of eCy3 fluorophores and the occurrence of two-step photodropping (Figure 5E). The corresponding time traces, occurrence probabilities and histograms of *T*_2_ for eCy3 labeled DNA molecules after the addition of 5.0 nM λ exonuclease are shown here (Figure 5E–G). A significant increase in the occurrence probability of two-step NISP events to ∼48.6% after the addition of λ exonuclease was observed, in comparison to 23.2% observed in the absence of λ exonuclease, indicating NISP assay can detect λ exonuclease-mediated ssDNA degradation process (Figure 5F and Table 4). A linear plot of *T*_2_ against the di-eCy3 labeling spacing (nt) yielded an intercept of 0.99 ± 0.10 s (Figure 5H, black squares), indicating that the eCy3 fluorophore causes hindrance, as corroborated by the bulk assay findings (Supplementary Figure S15). The derived slope from the linear fit (Figure 5H, black squares) indicates that λ exonuclease degrades phosphodiester bonds at an average rate of 0.083 ± 0.010 s nt^–1^. The ssDNA degradation rate of λ exonuclease is then deducible from the inverse of this slope in the linear regression (Figure 5H). After correcting for substrate length variations and subtracting the eCy3 influence, we calculated a degradation rate of 12.0 ± 1.5 nt s^–1^ for λ exonuclease in 5′-to-3′ directionality from the NISP assay (Figure 5H and Table 4).

**Table 4.** The probabilities distribution of the three scenarios in NISP determined 5′-to-3′ degradation rate of λ exonuclease on DNA substrates labeled with eCy3 fluorophores separated by different number of nucleotides with scissile phosphodiester bonds between the two Cy3 fluorophores (***S***)

		Probability (%)			
***S*** (nt)	[λ Exonuclease] (nM)	No photodropping	One-step NISP	Two-step NISP	*T*_2_ (s)
19	0	48.3 (198/410)	28.5 (117/410)	23.2 (95/410)	N.D.^a^
9	5	25.8 (31/120)	13.3 (16/120)	60.9 (73/120)	1.7 ± 0.8
14		26.1 (40/153)	17.0 (26/153)	56.9 (87/153)	2.2 ± 1.5
19		33.0 (133/403)	18.4 (74/403)	48.6 (196/403)	2.5 ± 2.0

^a^N.A., not assigned due to the low NISP probabilities.

## Discussion

In this report, we established a single-molecule method, named NISP, to investigate nuclease activity. The method offers a quick and convenient platform to access exonuclease activity at high temporal and spatial resolution. With the technique, the precise substrate-processing rate of nucleases can be measured without enzyme modifications, which can be laborious and time-consuming.

By NISP, we showed that MGME1 degrades ssDNA processively in both directions (Figures 1F and 3F), which was not resolved by previously reported conventional nuclease assays (22,23,27,29,30). No notable NISP signal was observed when using inhibitory ion or inactive MGME1-KH in the assay (Supplementary Figure S5), confirming that the NISP phenomena observed here are strictly dependent on the enzyme’s activity. By using blunt-end dsDNA as the substrate, we demonstrated that MGME1 lacks the ability to process duplex region (Figure 2), consistent with previous reports (22,23,27,31). Further, MGME1 made a higher two-step NISP occurrence probability on the 5′-ssDNA overhang substrates than the 3′-ssDNA overhang substrates (Table 1), indicating an overall better association of the enzyme with the 5′-ssDNA overhang, consistent with the previous study (30). Importantly, by comparing the two-step NISP occurrence probability induced by adding 1 nM MGME1, a ∼1.9-fold higher degradation efficiency of the enzyme in processing ssDNA from 5′ end than that from 3′ end can be calculated. We reasoned that a ∼3.1-fold faster degradation rate contributes to the higher degradation efficiency as MGME1 processing ssDNA from 5′ end (Figures 1 and 3). The precise measurement of such an asymmetric polarity for ss-specific exonuclease and endonuclease was not resolved in any previous analysis, neither by conventional biochemical assays nor single-molecule methods (11–17,22,23,27,29,30). According to our result, we propose a model in which MGME1 is an ssDNA-specific exonuclease with an asymmetric directionality that performs different degradation kinetics depending on whether the substrate DNA possesses a 3′- or 5′-ssDNA overhang (Figure 5A). The stronger 5′ polarity reported here and by others (22,30) agrees with the known biological role of MGME1 (22,24–28). Whether the 3′-exonuclease activity of MGME1 has a biological role may require further investigations.

Besides the implications we gain from the NISP result, it should be noted that the enzyme processing rates reported here were obtained under room temperature (22 ± 1°C), which could be underestimated since MGME1 presumably should work the best at the physiological condition (∼37°C). The implementation of a temperature-controlling system to the method may overcome the issue. Of note, based on the gel electrophoresis imaging analysis of bulk nuclease reaction done at 37°C, the previously published single-turnover kinetics data of MGME1 showed degradation of 7 nt in 6 s presumably from the 5′ end of a 10-nt, 3′-FAM-labeled poly(T) ssDNA (30). The estimated degradation rate was thus around ∼1.17 nt s^–1^, much slower than our measurement of 6.3 nt s^–1^ by NISP reported here.

**Figure 6.**
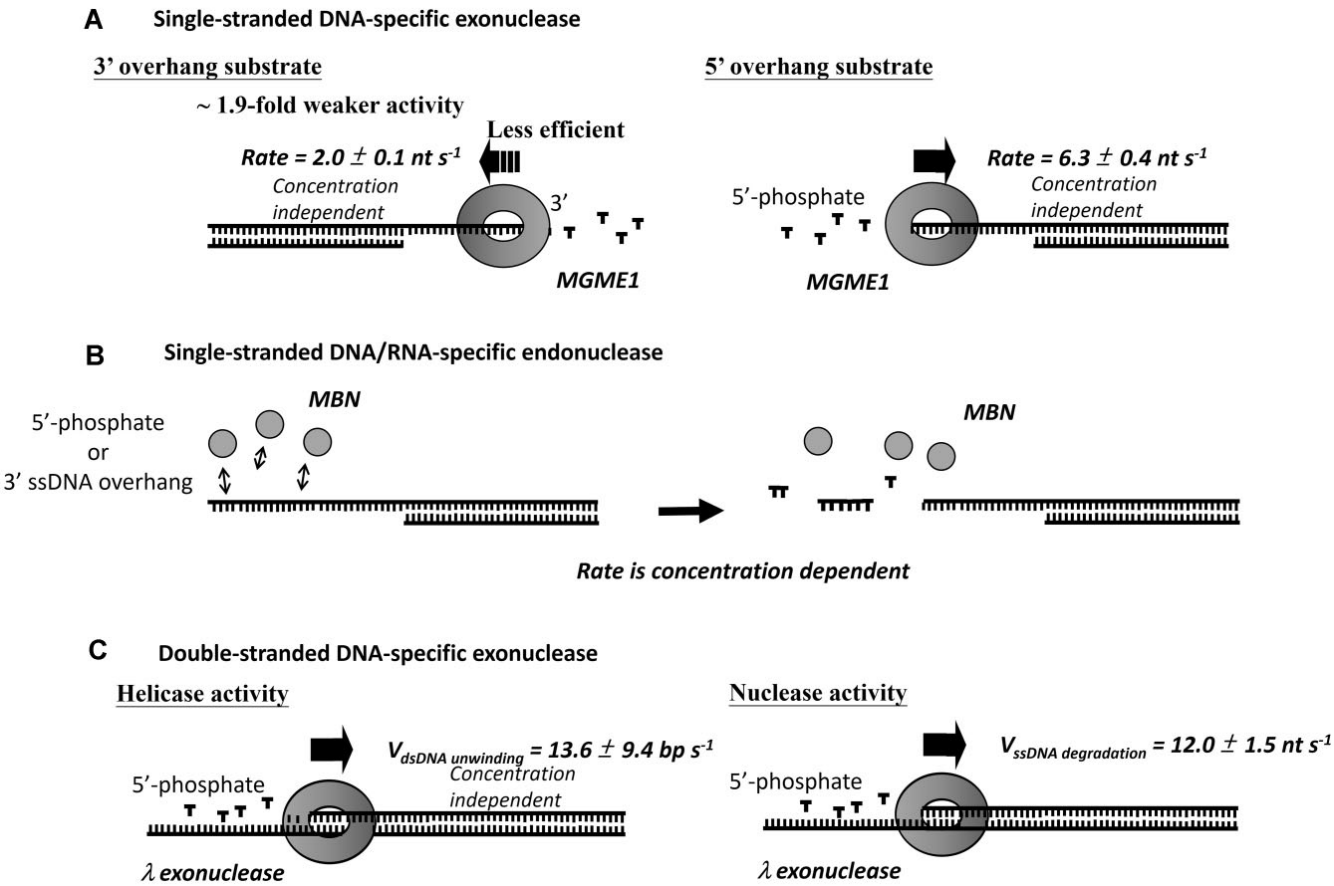
The kinetic model of the studied nucleases established by NISP in this work. (**A**) A kinetics model of MGME1-mediated ssDNA degradation. MGME1 can degrade ssDNA in either the 5′-to-3′ direction (right panel) or the 3′-to-5′ direction (left panel) in a processive manner. In this process, MGME1 exhibits a faster degradation rate when processing DNA from the 5′ end. (**B**) A kinetics model for MBN-mediated ssDNA degradation. MBN degrades ssDNA in a distributive manner. (**C**) A kinetics model for λ exonuclease-mediated DNA degradation. λ exonuclease can unwind dsDNA (left panel) and degrade one of the ssDNA (right panel) simultaneously in the 5′-to-3′direction.

Besides MGME1, we also applied NISP to investigate the activity of a commonly used nuclease, MBN. The enzyme is an ss-specific DNA/RNA endonuclease that can remove both 3′- and 5′-overhangs from dsDNA to create blunt ends (38,42). By conducting NISP experiments with various concentrations of MBN, we observed that the probabilities of both one-step and two-step NISP exhibited a concentration-dependent trend (Figures 4 and 6B; Table 3), which is entirely different from the NISP data obtained from MGME1. The data presented in this study demonstrate that the NISP assay can distinguish between distributive degradation and processive degradation behaviors. This capability allows the method to be applied for studying not only exonuclease but also endonuclease at the single-molecule level.

Lastly, NISP was used to investigate a commonly used dsDNA-specific exonuclease, λ exonuclease. In previous studies, the catalytic activities of λ exonuclease have been studied with bulk biochemistry assays and single-molecule approaches to obtain the processive degradation rates of 10–12 nt s^–1^ (43,44) and 13–18 nt s^–1^ (39,8,6,41) respectively. Moreover, it has been pointed out that λ exonuclease might unwind the dsDNA substrate before the process of ssDNA cleavage (45). The abovementioned techniques might precisely determine the dsDNA unwinding rate instead of ssDNA degradation rate for λ exonuclease. In this work, the dsDNA unwinding rate of 13.6 ± 9.4 bp s^–1^ obtained from FRET and ssDNA degradation rate of 12.0 ± 1.5 nt s^–1^ obtained from NISP for λ exonuclease can be separately and precisely determined (Figures 5 and 6C; Table 4). The similar rates observed for λ exonuclease-mediated ssDNA degradation and dsDNA unwinding are consistent with the mechanism in which λ exonuclease catalyzes dsDNA unwinding prior to ssDNA degradation (45). NISP offers an alternative method to assess λ exonuclease activity, focusing on the removal of ssDNA strands rather than the melting of the duplex region, as measured in previous FRET studies. Comparatively, NISP provides more precise data, as indicated by a lower standard deviation (SD_NISP_ = 1.5 versus SD_FRET_ = 9.4). Moreover, while FRET is limited to an application range of 1–10 nm, NISP, by labeling di-Cy3 at specific intervals along ssDNA substrates, offers a broader application range. Furthermore, for nucleases with rapid degradation rates, FRET may fail to accurately measure ssDNA degradation due to the lack of detectable FRET signals when the Cy3–Cy5 pair spacing exceeds 10 nm, especially with longer DNA substrates. NISP, however, does not encounter this limitation, making it a superior choice for studying fast-acting nucleases.

In conclusion, we introduce a simple, convenient and protein-modification-free method to investigate nuclease degradation behaviors at the single-molecule level. The NISP assay precisely determines substrate preferences and nuclease processivity, enhancing our knowledge of nuclease kinetics. Based on bulk assay data regarding substrate preferences and the effects of Cy3 fluorophore labeling on nuclease degradation, we can design appropriate DNA substrates for the NISP assay. Measuring *T*_2_ on DNA substrates labeled with di-Cy3 at various intervals (***S***), and plotting these *T*_2_ values against ***S***representing the number of nucleotides with scissile phosphodiester bonds between the two Cy3 fluorophores, enables the accurate calculation of the enzyme’s degradation rate by differentiating *T*_2_ into *T*_2,exo_ and *T*_2,Cy3_ components, which are obtained from the slope and intercept of the linear plot, respectively. Regardless of the diverse ways of binding the DNA backbone during subsequent exonucleolytic cuts from either the 3′ or 5′ end, the interference of Cy3 labeling can be extracted from the intercept with a linear plot. For nucleases with lower processivity, it is advisable to use a duplex DNA with an ssDNA overhang shorter than its processivity. Moreover, the spacing between di-Cy3 fluorophores should be longer than the degradation rate (nt s^–1^) multiplied by the time resolution of the imaging CCD used in the acquisition system. In the future, by labeling more than two Cy3 fluorophores, such as tri-Cy3 or tetra-Cy3, at known intervals, length-dependent *T*_2_ values can be simultaneously obtained from a single NISP experiment. This approach mitigates time constraints and enables the rapid acquisition of degradation information. The NISP method is versatile and can be applied to study a wide range of nucleases, providing critical insights into their kinetics and expanding our understanding of these essential biological components.

## Supplementary Material

gkae822_Supplemental_File

## Data Availability

The data underlying this article will be shared on reasonable request to the corresponding author.
