# Supplementary material for: Nuclease-induced stepwise photodropping (NISP) to precisely investigate single-stranded DNA degradation behaviors of exonucleases and endonucleases
Source: Nucleic Acids Res. 2024 Oct 1;52(20):e97. doi: 10.1093/nar/gkae822 (PMC11551736; doi:10.1093/nar/gkae822)
Supplement: gkae822_Supplemental_File [file gkae822_supplemental_file.pdf]

Supplementary information
for
Nuclease-induced stepwise photodropping (NISP) to precisely investigate single-stranded DNA degradation behaviors of exonucleases and endonucleases

Hui-Pin Chiu[1,2+], Chung-Han Shen [1,2+], Jan-Kai Wu[1,2], Eric Y.C. Mao[3], Han-Yi Yen[3], Yuan-Pin Chang[2], Chyuan-Chuan Wu[3,*], Hsiu-Fang Fan[1,2,4,*]

[1] Institute of Medical Science and Technology, National Sun Yat-sen University, Taiwan
[2] Department of Chemistry, National Sun Yat-sen University, Taiwan
[3] Department of Biochemistry and Molecular Biology, College of Medicine, National Cheng Kung University, Taiwan

[4] Lead contact

Corresponding Author
*Hsiu-Fang Fan, E-mail: bendyfan@imst.nsysu.edu.tw
*Chyuan-Chuan Wu, E-mail: ccwu@gs.ncku.edu.tw
+: These authors have equal contributions to this work

Supplementary Text

Supplementary Methods

Supplementary Figures S1 to S15

Supplementary Tables S1

Supplementary References

Supplementary Text

The analysis of MGME1-derived cleavage pattern.

Using the oligonucleotide probes with the same DNA sequences but without Cy3 modification in the ssDNA region, we examined MGME1's nuclease activity on 5′- or 3′-overhang ssDNA with the conventional bulk nuclease assay. The scissile strands were end-labeled with either Cy5 or FAM for tracking the MGME1-derived cleavage products in gel electrophoresis (Supplementary Figure S11). In a time-course experiment done in parallel, our data revealed that MGME1 is more efficient in processing 5′ ssDNA overhang, as evidenced by the fewer cleavage intermediates spanning the ssDNA region and a faster accumulation of cleavage products around the ssDNA-dsDNA junction (Supplementary Figure S11 A and C). In contrast, the 3′ overhang substrate was degraded gradually over time from the 3′ end and the cleavage pattern kept developing until the last time point (70 minutes) (Supplementary Figure S11 B and D). Further, our gel imaging also showed that MGME1 would obligatory leave short (3-to-4 nt) 5′ overhang unresolved when processing DNA for 5′ ends, whereas cleave into the duplex region for around 3 nt when processing in the other direction. The phenomenon was explained by the structural analysis of substrate-bound MGME1 complexes(1,2). These structures reveal that the catalytic center resides "upstream" (*i.e.*, at 5′ side according to the bound substrate) of the helical arch of the enzyme for about 3-to-4 nt. Since MGME1 would stop DNA translocation when the helical arch reaches the duplex region, this misalignment between the catalytic center and the ssDNA-selective helical arch would obligatorily result in a short 5′ overhang on either scissile (when processing from 5′ end) or non-scissile strand (when processing from 3′ end) unresolved on the DNA substrates.

To evaluate the effect of iCy3 and eCy3 modifications on MGME1's activity, we used the bulk nuclease assay to inspect the DNA cleavage pattern derived by the enzyme (Supplementary Figure S2). Due to the limited DNA separating resolution of the products derived from the 58-nt DNA in the gel-based imaging, here, we employed a collection of shorter (with a 28-nt scissile strand) DNA substrates with only a single iCy3 or eCy3 labeled in the 14-nt ssDNA overhang regions (Supplementary Figure S2 A and C). The resultant cleavage pattern of the Cy3-labeled 5′-overhang DNA well agrees with our previous analysis(2), revealing that MGME1 cut at the 5′ side of the Cy3 fluorophore, therefore generating cleavage intermediates above the Cy3 site (indicated by arrowheads in Supplementary Figure S2B) in the gel analysis. As for the cleavage pattern of the Cy3-labeled 3′-overhang DNA, we surprisingly found that MGME1 mainly cut at the 3′ sides of the fluorophores, as indicated by the persistent Cy3 signal of the cleavage intermediates (Supplementary Figure S2D). This result differs from our previous analysis by using a 58-nt, di-eCy3-labeled substrate, where

the second intermediate cluster had no Cy3 signal, indicating that MGME1 cut at the 5′ side of eCy3, therefore removing the fluorophore from the intermediates. We reasoned that the inconsistency may be due to the substantially shorter 3′-overhang region that we used in this study, but why exactly MGME1 would behave differently on the shorter overhang requires further investigation. It also should be noted that multiple cleavage products carrying the Cy3 fluorophore were observed as MGME1 digesting the ssDNA overhang, no matter which digesting directions (see the gel images signaled by Cy3 in Supplementary Figure S2 B and D), indicating that more than one nucleotide could be removed along with the Cy3 fluorophore by the enzyme. Given that MGME1 essentially works as a DNA clamp when translocating on ssDNA, we reasoned that MGME1 may skip cleavable bonds when encountering the bulky Cy3 fluorophore which could impose steric hindrance to the enzyme's action.

The analysis of mung bean nuclease (MBN) and λ exonuclease-derived cleavage patterns.

Following our examination of MGME1, to evaluate the effect of iCy3 and eCy3 modifications on the activities of MBN and λ exonuclease, we used the bulk nuclease assay to inspect the DNA cleavage pattern (Supplementary Figures S14 and S15).

For MBN, the same collection of 14-nt overhang DNA used for MGME1 bulk assay was applied for the investigation (Supplementary Figures S14 A and C). We found that MBN exhibits none-specific endonuclease activity toward the 14-bp duplex region of the substrates, revealed by the cleavage products shorter than the 14-nt marker (M2) when incubated the DNA with 1 U MBN for 5 minutes (lanes 3, 6, and 9 in Supplementary Figure S14 B and D). Therefore, we reduced the enzyme amount to 0.1 U and found that MBN mostly cleaved at the ssDNA overhang regions and generated the end product of 14 nt (lanes 2, 5, and 8). The varied sizes of cleavage products carrying Cy3 signal (see Cy3 images) reveal that MBN cleaves the ssDNA overhang region endonucleolytically, and the cleavage positions were somewhat altered by eCy3 and iCy3 modifications (comparing lanes 2, 5, and 8 in the Cy5 images). Nevertheless, as an endonuclease, MBN's efficiency in consuming the substrate was not significantly affected by either eCy3 or iCy3 modifications. We then chose to use eCy3-labeled DNA for assaying the enzyme's activity in smNISP.

For λ exonuclease, 28-bp dsDNA substrates with or without Cy3 modifications were prepared (Supplementary Figures S15A). The result showed that iCy3 modification blocks the enzyme from digesting the DNA to the end product (P), indicated by the accumulation of cleavage product around the iCy3 site (lane 4 in Supplementary Figure S15B). In contrast, the enzyme can generate the end product from the eCy3-labeled substrate (lane 6) as well as it digests the label-free substrate, with only a minor accumulation of the cleavage intermediates around the eCy3 site.

Accordingly, we concluded that eCy3 does not significantly influence λ exonuclease-mediated DNA degradation and chose to use eCy3-labeled dsDNA for assaying λ exonuclease's activity in smNISP.

Supplementary Methods

Molecular cloning

The coding region of human MGME1 (without mitochondrial-targeting sequence; base pair (bp) 61 to 1,032) was cloned into the pSol-SUMO expression vector following the manufacturer's instruction (Lucigen). The generated pSol-SUMO-MGME1 plasmid was used as the template in site-directed mutagenesis to introduce two additional His codons in the N-terminal His6-tag, generating the pSol-H8-SUMO-MGME1 plasmid. H180A and K253A substitutions were introduced to the plasmid by site-directed mutagenesis, resulting in the pSol-H8-SUMO-MGME1-KH plasmid. Primer sequences for the mutagenesis are listed below with the mutated codons underlined: H180A-forward: 5′-ACGGTTC<u>GCC</u>GAAGCCTTGGAAAGC-3′; H180A-reverse: 5′-AGGCTTC<u>GGC</u>GAACCGTTTCCCTTG-3′; K253A-forward: 5′-GATTGATTGG<u>GCG</u>ACATCAGAGAAACCAAAGCC-3′; K253A-reverse: 5′-TCTGATGT<u>CGC</u>CCAATCAATCACACAGAGCTTG-3′.

In vitro nuclease assay

To compare the 5′- and 3′-exonuclease activities of MGME1 (result shown in Supplementary Figure S11), synthetic oligonucleotide probes with either 3′-Cy5 or 5′-6-carboxyfluorescein (FAM)-labeling (Genomics, Taiwan) were used to prepare 5′-overhang and 3′-overhang DNA substrates, respectively. The sequence of the probes is identical to that used in single-molecule NISP assay (Supplementary Table S1). 5′- and 3′-overhang DNA substrates were prepared by annealing with complementary strands in an annealing buffer (10 mM HEPES pH 7.4, 50 mM NaCl). 100 nM of the substrate was incubated with serial diluted MGME1 in a 10 μL reaction containing 100 μg mL^{-1} bovine serum albumin (BSA), 10 mM HEPES pH 7.4, 150 mM NaCl, and 2.5 mM $MgCl_2$ at 37°C. After the desired incubation time, reactions were stopped by adding 10 μL of 2× TBE/urea sample buffer (G-Biosciences) and heating at 65°C for 20 minutes. To fully release the probe from the complementary strand, 2 μL of 100 μM competitive DNA was added (Supplementary Table S1), and the resultant mixtures were heated at 95°C for 10 minutes and gradually cooled to 4°C. The samples were separated with a 20% native TBE acrylamide gel. Signals from Cy5- and FAM-labeled probes in the resultant gels were visualized using a Typhoon FLA 9000 biomolecular imager (GE Healthcare Life Sciences), with excitation/emission at 635 nm/670 nm for Cy5 and 488 nm/525 nm for FAM. Band intensity was quantified by Image J(3).

To evaluate the effect of iCy3 and eCy3 modifications on the nuclease activity of MGME1, MBN and λ exonuclease, a collection of DNA substrates having 14-nt scissile strands without or with a single Cy3 modification was prepared (Supplementary Table S1). For MGME1, the experiments with the 14-nt overhang DNA substrates were done following the protocol described in the previous paragraph with minor modifications. First, the 2× TBE/urea sample buffer was replaced with a dye-free version of the buffer to avoid interfering with Cy5 signal imaging. Second, the cleavage products were separated with 20% denaturing TBE acrylamide gels containing 7 M urea. Third, the Cy3 signal (excitation/emission at 532 nm/570 nm) was recorded to reveal if the cleavage products carry the Cy3 fluorophore. The results are shown in Supplementary Figure S2. Band intensity signaled by Cy5 was quantified by Image J (3).

For MBN and λ exonuclease (both were purchased from New England Biolabs), the experiments were done according to the manufacture's instructions. In brief, a 10 μL reaction mixture containing 100 nM DNA substrate, 1× reaction buffer, and the enzymes were incubated at the recommended temperature. For MBN, the reaction was incubated for 5 minutes and stopped by supplying 0.1% SDS to the mixture. For λ exonuclease, the reaction was incubated for one minute and stopped by supplying 10 mM EDTA to the mixture and heating at 75°C for 10 minutes. Probe displacement and gel electrophoresis were done with the same procedure described above. The results are shown in Supplementary Figures S14 and S15.

Supplementary Figures and Tables

(A)

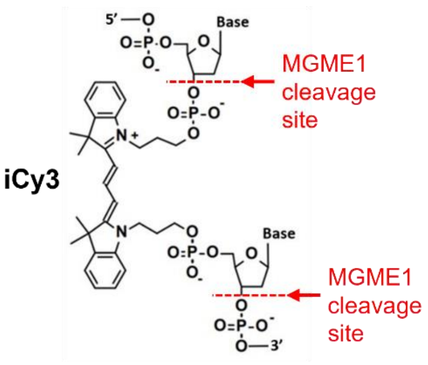

(B)

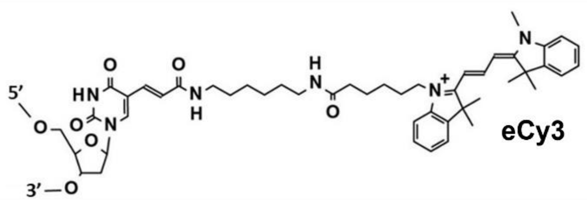

Supplementary Figure S1. *The structure of Cy3 fluorophores used to label DNA substrates in this study.* **A.** The structure of internal Cy3 (iCy3). **B.** The structure of external Cy3 (eCy3).

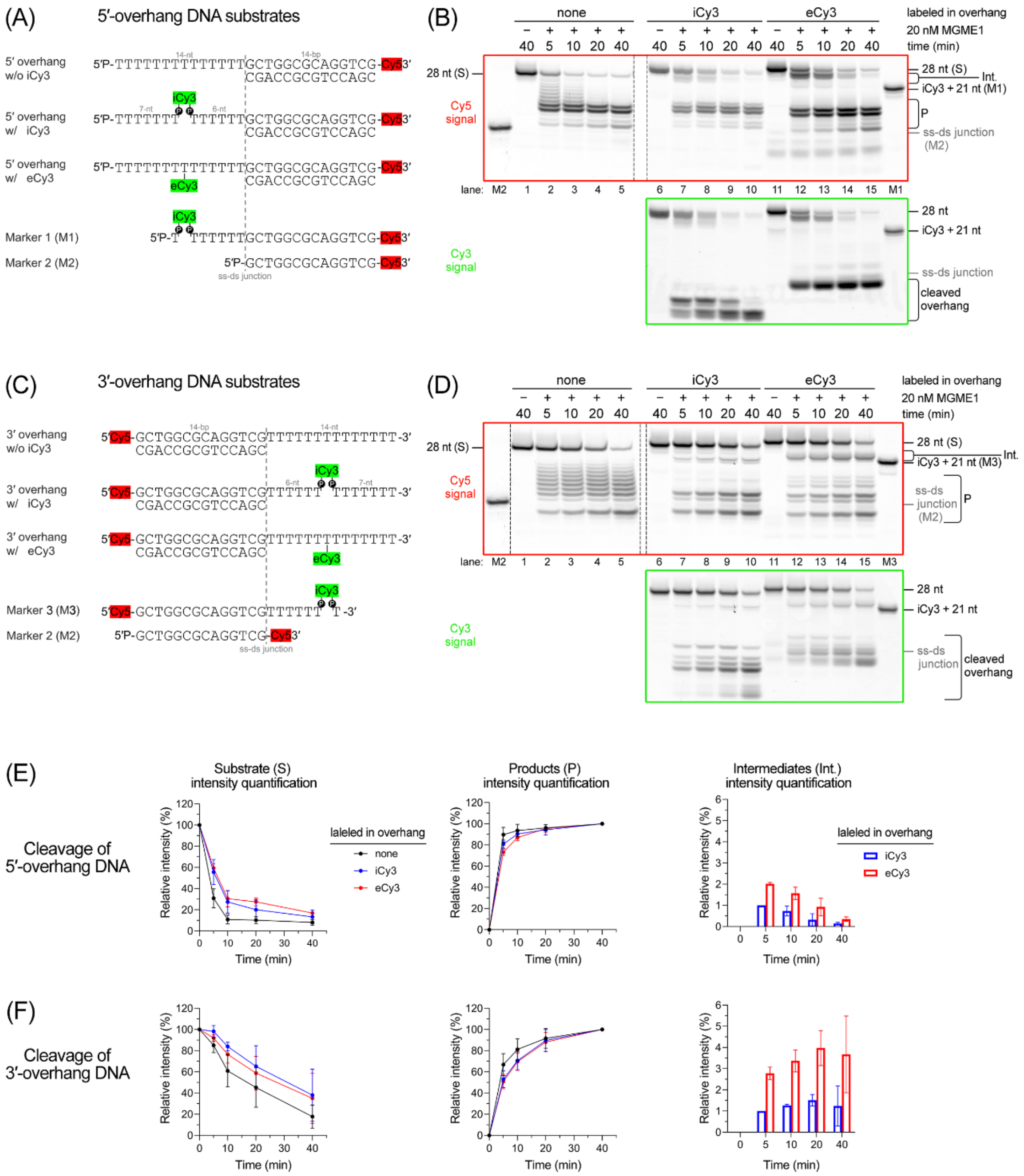

Supplementary Figure S2. *In-vitro nuclease assay of MGME1 in degrading Cy3-labeled overhang DNA*. The design of the substrate is shown in panels **A** and **C**. **B** and **D.** MGME1 in degrading the 14-nt 5′-overhang (**B**) and 3′-overhang (**D**) DNA. The reaction was done according to the description in Supplementary Methods. Note that the Cy5-labeled scissile strand was displaced from the complementary strand and separated with a 20% TBE acrylamide gel containing 7 M urea. **E** and **F**. Quantification of MGME1-derived DNA cleavage pattern. Band intensity of substrate (S), cleavage products (P), and the Cy3-induced cleavage intermediates (Int.) was quantified and plotted against reaction time.

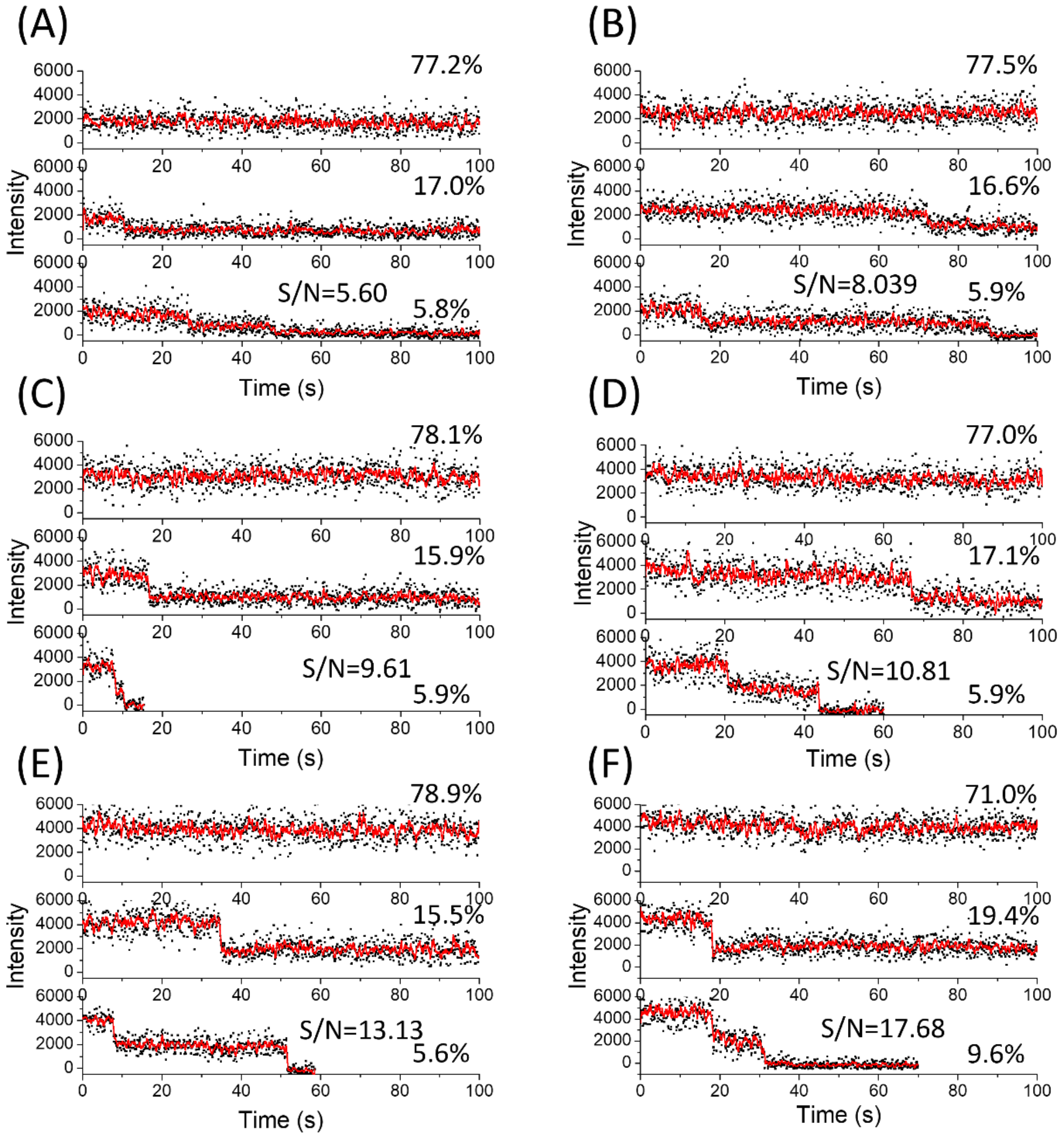

Supplementary Figure S3. *Influence of illumination intensity on spontaneous photodropping behaviors*. This figure shows the event probability of no photodropping, one-step photodropping, and two-step photodropping for DNA molecules with a 5′-phosphorylated poly d(T)$_{40}$ ssDNA overhang labeled with two iCy3 fluorophores, with a 18-nt interval in between. The experiments were conducted under different illumination intensities: **A.** 3mW, **B.** 4mW, **C.** 5mW, **D.** 6mW, **E.** 7mW, **F.** 8mW. (Similar trends were observed with eCy3 fluorophores, although data are not shown).

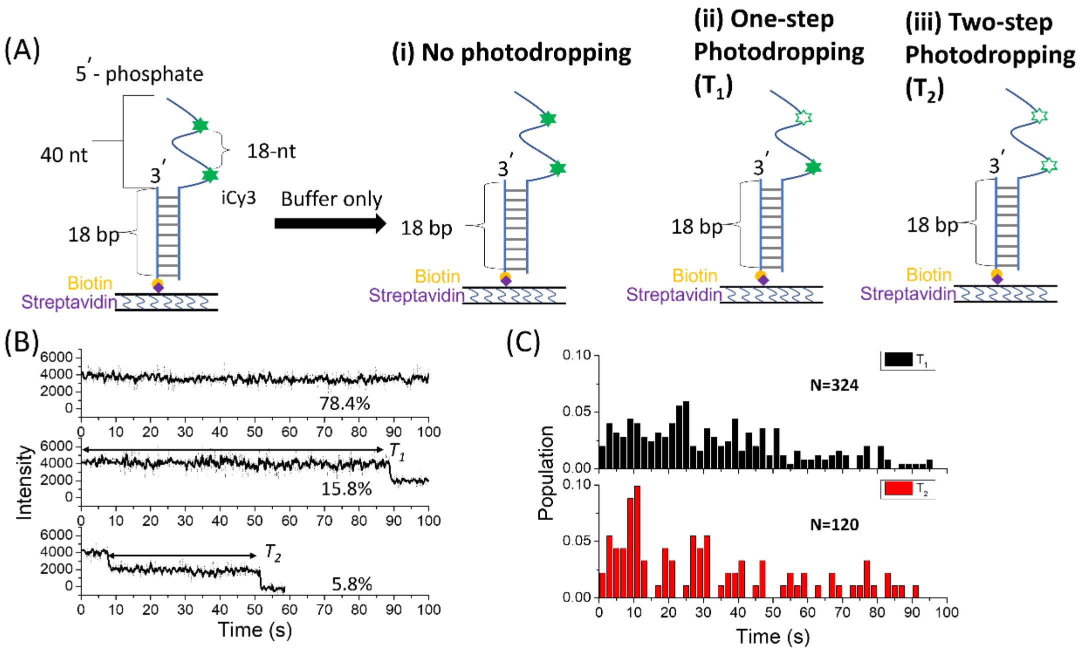

Supplementary Figure S4. *NISP control experiments on a DNA with a 5'-phosphorylated poly d(T)$_{40}$ ssDNA overhang labeled with two iCy3 fluorophores with a 18-nt interval in between.* **A**. A DNA named 5’→3’ di-iCy3-18nt was used. After buffer injection, three scenarios were observed: (i) No detectable photodropping, (ii) One-step photodropping, and (iii) Two-step photodropping. **B**. Typical NISP time traces illustrating the behaviors observed in **A** are shown. The dwell time in the one-step photodropping time trace (indicated by a double-arrowed line) is named T_1, and the dwell time marked with a double-arrowed line in the two-step photodropping time trace is named T_2. The values indicate the occurrence probabilities determined from 2,055 analyzed molecules (Table 1). **C**. Histograms of the observed T_1 and T_2 in the absence of MGME1. Green asterisks represent iCy3 fluorophores.

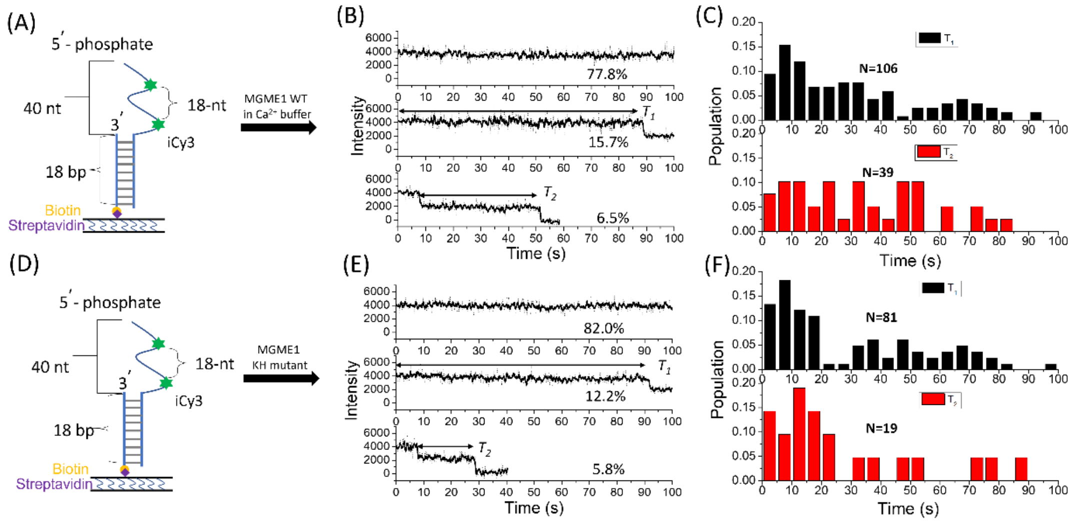

Supplementary Figure S5. *MGME1 failed to produce NISP signal in the presence of Ca^{2+} or when the catalytic residues were mutated.* **A**. Schematic of the NISP assay with MGME1 in the presence of Ca^{2+} ions. A DNA named 5’→3’ di-iCy3-18nt dsDNA was used. **B**. The NISP time traces resulted from panel A. **C**. Histograms of the observed T_1 and T_2 in the presence of 5.0 nM MGME1 within a Ca^{2+} ion-containing reaction solution. **D**. Schematic of the NISP assay with MGME1-KH. A DNA named 5’→3’ di-iCy3-18nt dsDNA was used. **E**. The NISP time traces resulted from panel D. The dwell time in the one-step NISP time trace (indicated by a double-arrowed line) is named T_1, and the dwell time marked with a double-arrowed line in the two-step NISP time trace is named T_2. **F**. Histograms of the observed T_1 and T_2 in the presence of 5.0 nM MGME1-KH. The dwell time in the one-step NISP time trace (indicated by a double-arrowed line) is named T_1, and the dwell time marked with a double-arrowed line in the two-step NISP time trace is named T_2. N indicates the event number. Green asterisks represent iCy3 fluorophores.

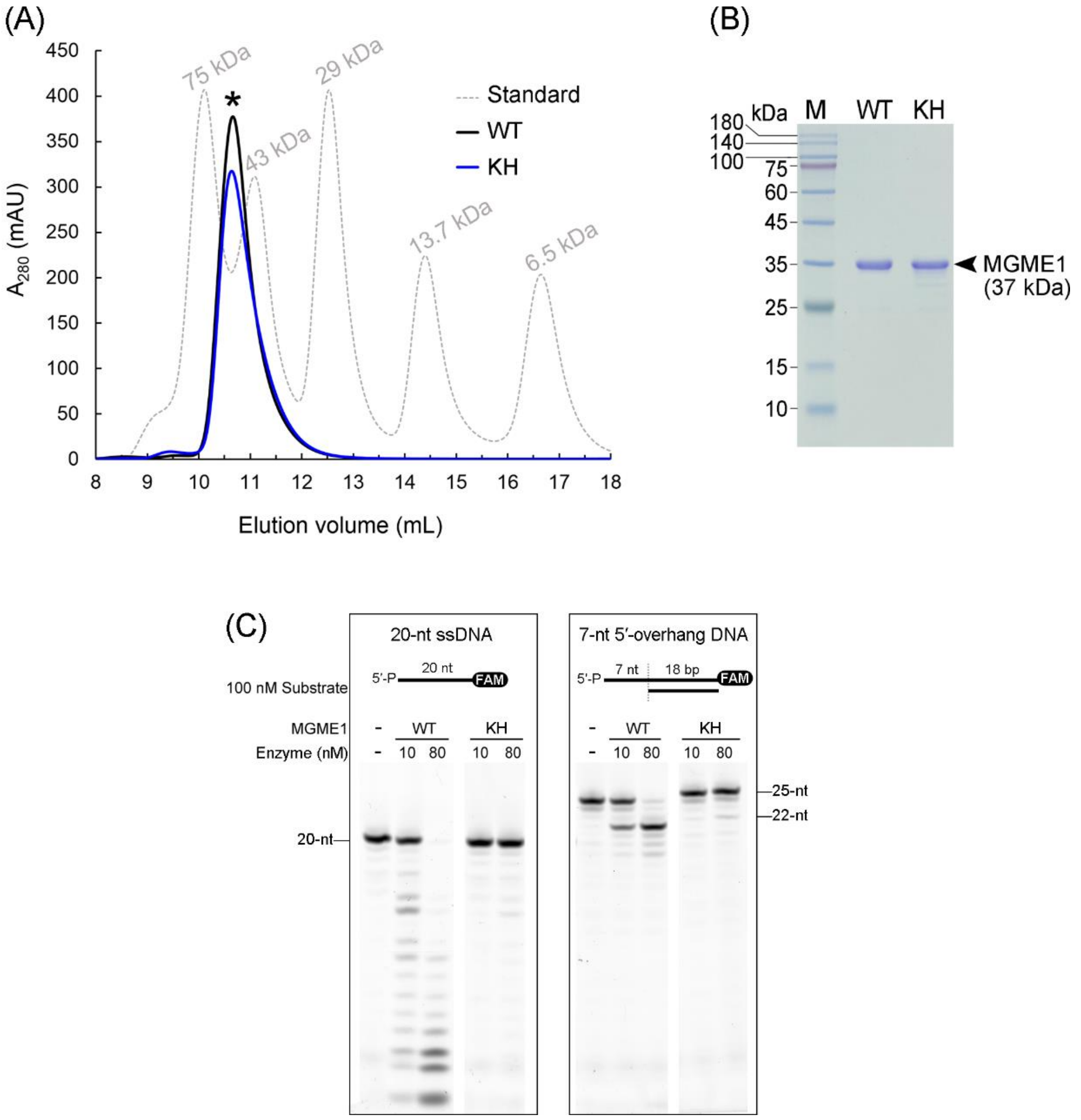

Supplementary Figure S6. *Purification of MGME1 proteins used in this study and nuclease activity test of MGME1-KH.* **A**. Analytical size-exclusion chromatography (SEC) of the purified MGME1 proteins. Superdex 75 Increase 10/300 GL column was used for the analysis. Standard proteins are: Conalbumin (75 kDa), Ovalbumin (43 kDa), Carbonic anhydrase (29 kDa), Ribonuclease A (13.7 kDa) and Aprotinin (6.5 kDa). **B**. SDS-PAGE of the purified MGME1 proteins. The gel was stained with Coomassie blue following standard protocol. The calculated molecular weight of the recombinant MGME1 is approximately 37 kDa. **C**. *In-vitro* nuclease activity test of MGME1-KH. The experiment was done according to Supplementary Methods. The reaction time for digesting ssDNA and 5′-overhang DNA was 5 and 10 minutes, respectively. Sequence of the probes are listed in Supplementary Table S1.

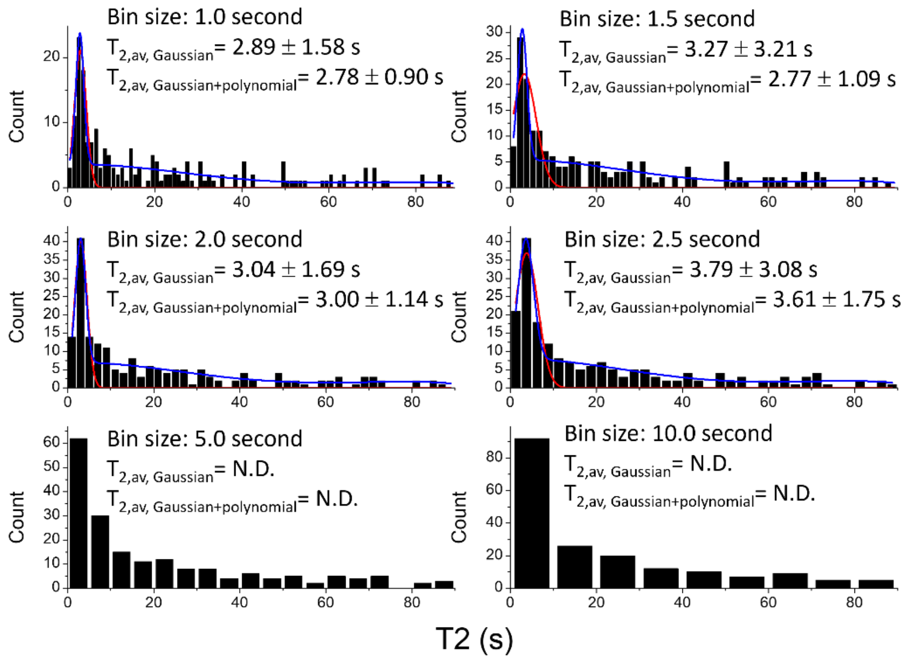

Supplementary Figure S7. *Evaluation of T_2 histogram bin size.* Histograms depict the T_2 distribution under 10nM MGME1 presence, with varying bin sizes. The red line represents the fit to a Gaussian distribution without considering spontaneous photodropping, while the blue line shows the fit to a Gaussian distribution along with a quartic polynomial function serving as the background for spontaneous photodropping. The values displayed indicate the average degradation times ($T_{2,\ av}$). It can be observed that the fitted values remain relatively constant when the binning size is smaller than the fitting value. The maximum suitable binning size is determined based on when the obtained fitted value does not significantly deviate from others. In the current case, the binning size is set at 2 seconds. Red lines represent the Gaussian fittings and blue lines represent the Gaussian plus polynomial fittings.

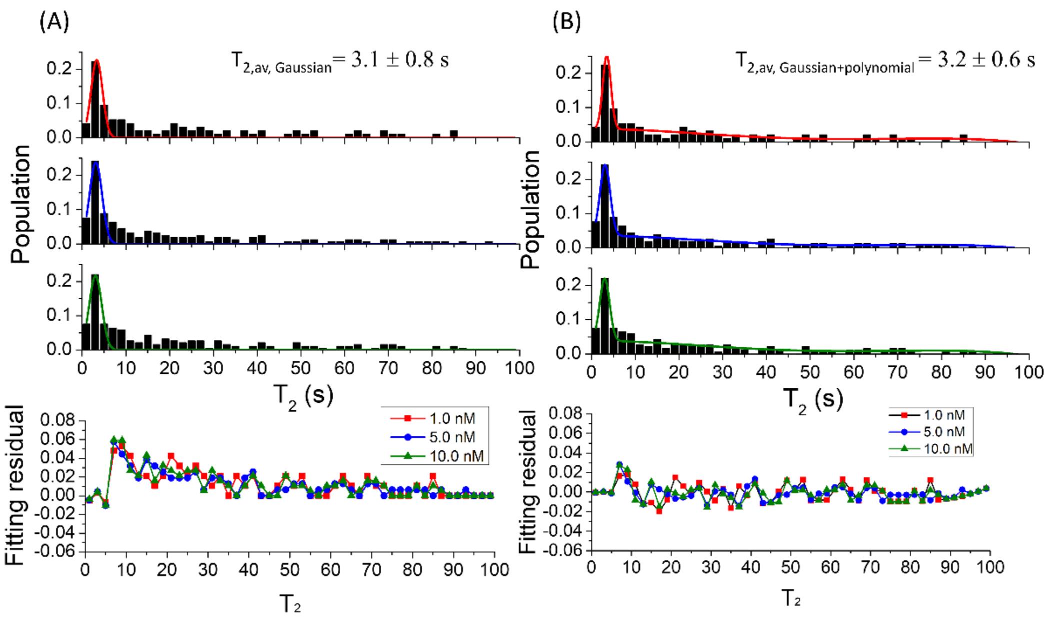

Supplementary Figure S8. *Evaluation of the fitting algorithm.* **A**. Without background subtraction: the distribution of T_2 in the presence of various concentrations of MGME1 fitted to a Gaussian distribution without considering spontaneous photodropping. **B**. With background subtraction: the distribution of T_2 in the presence of various concentrations of MGME1 fitted to a Gaussian distribution, along with a quartic polynomial function serving as the spontaneous photodropping background. The curves represent the fitting results, and the fitting residual is shown below. The values shown here are the average degradation times ($T_{2,\ av}$).

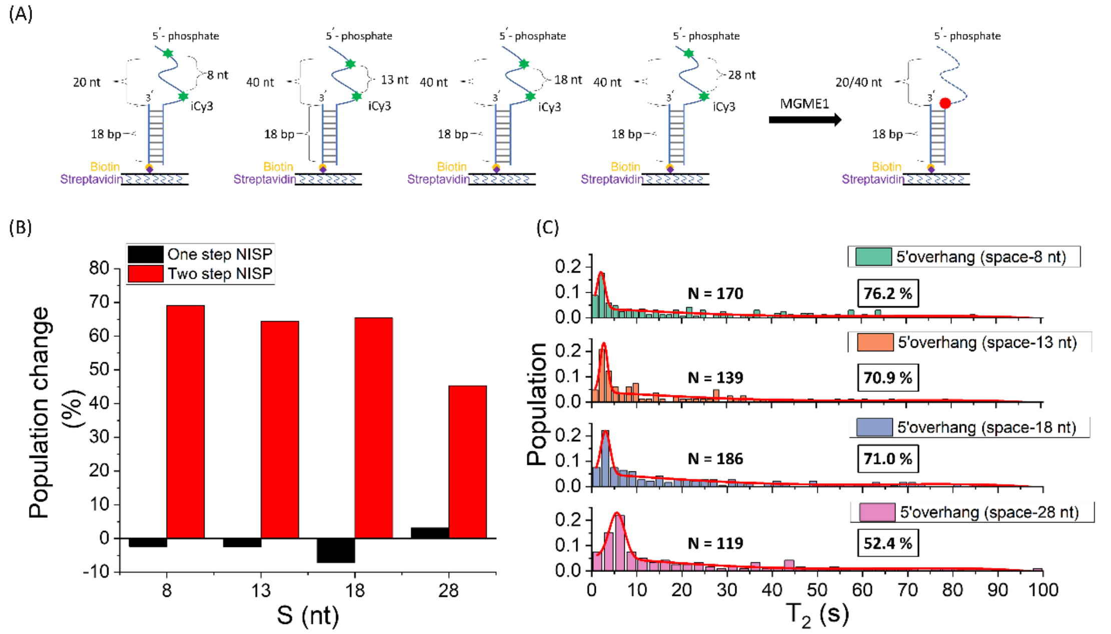

Supplementary Figure S9. *Length dependence of MGME1-mediated ssDNA degradation from 5' to 3' direction on di-iCy3 labeled DNA substrates.* **A**. Schematic illustrating of NISP for investigating ssDNA degradation by exonuclease, with a DNA substrate featuring a 5′-phosphorylated poly $(dT)_n$ overhang (n=20,40) anchored on a PEGylating slide. The numbers of nucleotides with scissile phosphodiester bonds between two iCy3 fluorophores are 8, 13, 18 and 28 nt. After injection of exonuclease, ssDNA degradation can be detected by the stepwise photodropping of iCy3 signals. Green asterisks represent iCy3 fluorophores and red circles represent MGME1 nucleases. **B**. The plots of population change of one-step and two-step NISP. **C.** Histograms of T_2 of MGME1 in degrading DNA substrate featuring a 5′-phosphorylated poly $(dT)_n$ overhang (n=20,40). Red lines indicate the nonlinear polynomial plus Gaussian fitting for T_2 distribution (in the presence of MGME1). These determined T_2 values are listed in Table 2.

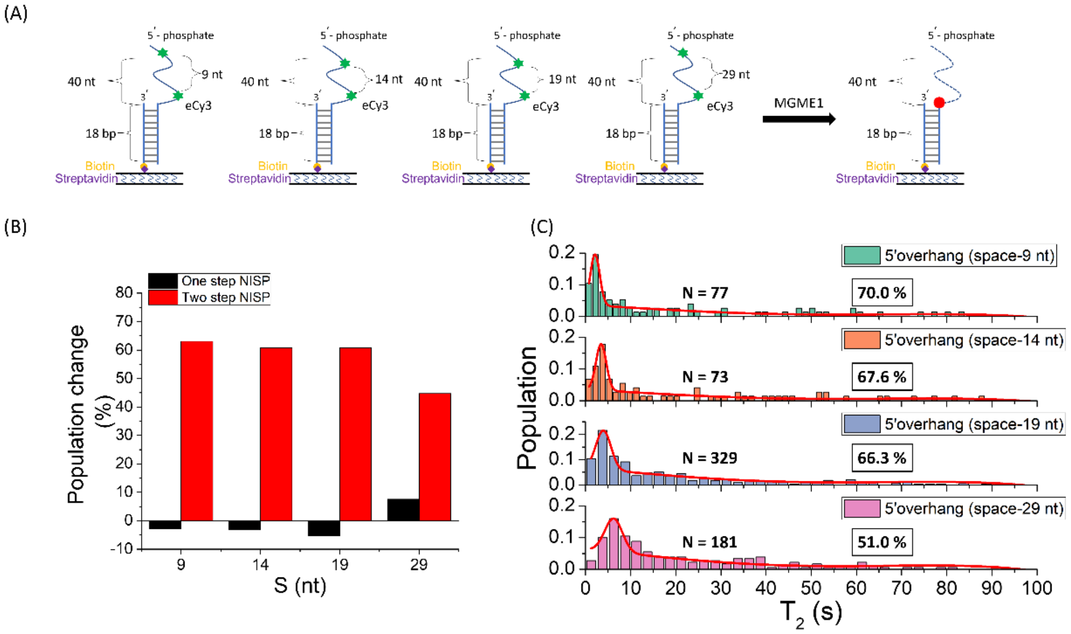

Supplementary Figure S10. *Length dependence of MGME1-mediated ssDNA degradation from 5' to 3' direction on di-eCy3 labeled DNA substrates.* **A**. Schematic illustrating of NISP for investigating ssDNA degradation by exonuclease, with a DNA substrate featuring a 5′-phosphorylated poly $(dT)_{40}$ overhang anchored on a PEGylating slide. The numbers of scissile phosphodiester bonds between two eCy3 fluorophores are 9, 14, 19, and 29 nt. After injection of exonuclease, ssDNA degradation can be detected by the stepwise photodropping of eCy3 signals. Green asterisks represent eCy3 fluorophores and red circles represent MGME1 nucleases. **B**. The plots of population change of one-step and two-step NISP. **C.** Histograms of T_2 of MGME1 in degrading DNA substrate featuring a 5′-phosphorylated poly $(dT)_{40}$ overhang. Red lines indicate the nonlinear polynomial plus Gaussian fitting for T_2 distribution (in the presence of MGME1). These determined T_2 values are listed in Table 2.

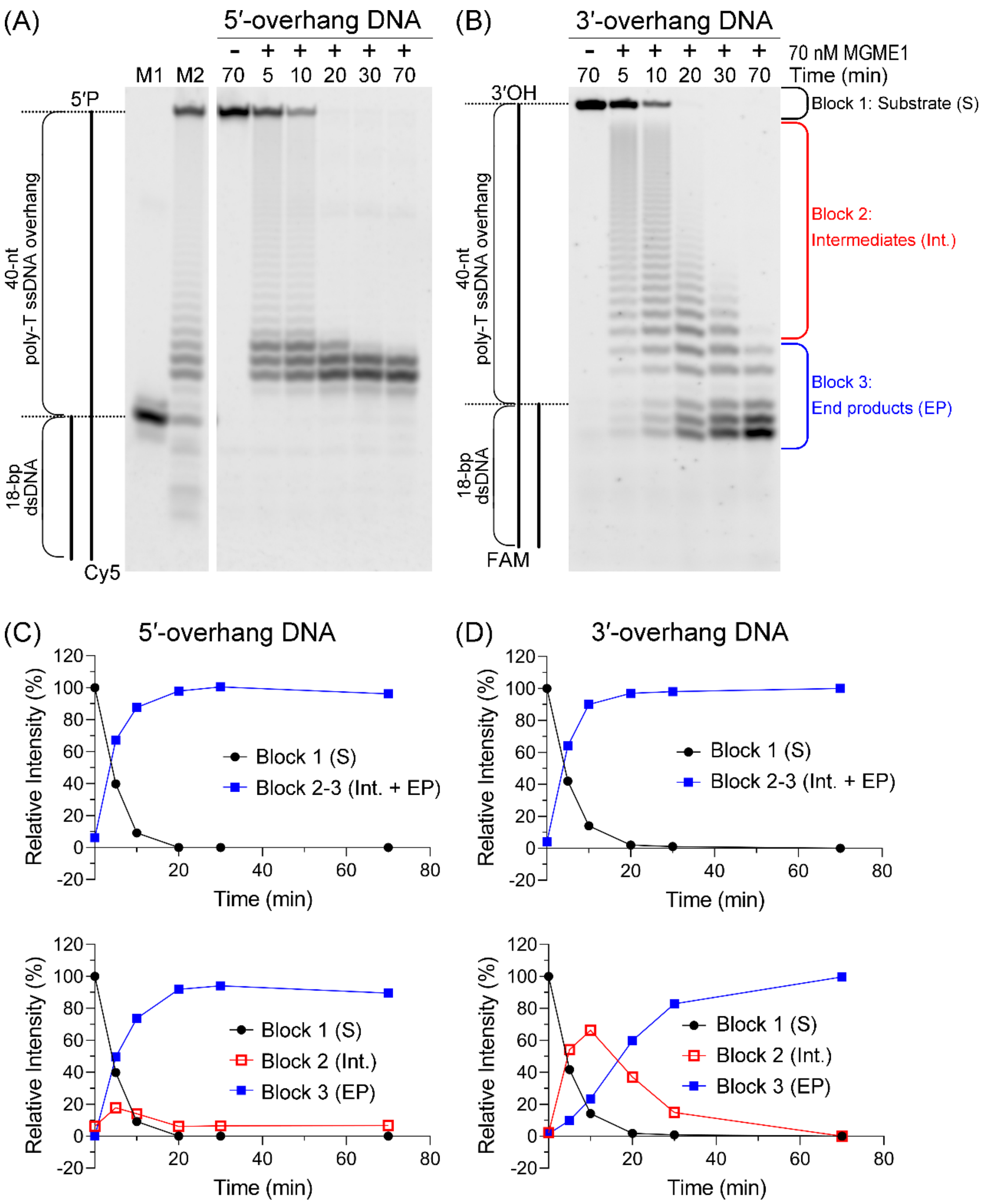

Supplementary Figure S11. *In-vitro nuclease assay reveals the higher efficiency of MGME1 in degrading ssDNA from the 5′ end.* **A** and **B**. MGME1 in digesting the 40-nt 5′-overhang (**A**) and 3′-overhang (**B**) DNA. Marker 1 (M1) is a synthetic 18-nt ssDNA labeled with 3′-Cy5, having the same sequence as the 3′-terminal 18 nt of the scissile strand of the 5′-overhang DNA substrate (see Supplementary Table S1). Marker 2 (M2) is a mixture of M1 and the reaction products of MGME1 digesting the single-stranded scissile strand of the 5′-overhang DNA substrate. The reaction was done according to the description in Supplementary Methods. Note that the Cy5- or FAM-labeled scissile strand was displaced from the complementary strand and separated with a native 20%

TBE acrylamide gel. **C** and **D.** Quantification of the substrate (S), cleavage intermediates (Int.), and end products (EP) from panels **A** and **B**, respectively.

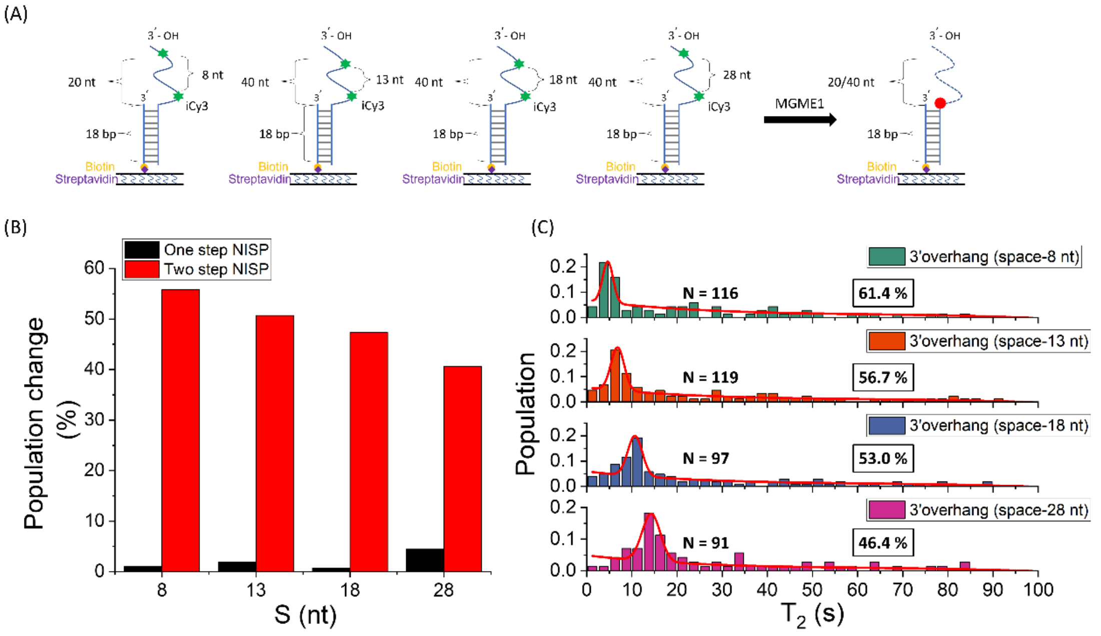

Supplementary Figure S12. *Length dependence of MGME1-mediated ssDNA degradation from 3' to 5' direction on di-iCy3 labeled DNA substrates.* **A**. Schematic illustrating of NISP for investigating ssDNA degradation by exonuclease, with a DNA substrate featuring a 3′ poly $(dT)_n$ overhang (n=20,40) anchored on a PEGylating slide. The numbers of scissile phosphodiester bonds between two iCy3 fluorophores are 8, 13, 18, and 28 nt. After injection of exonuclease, ssDNA degradation can be detected by the stepwise photodropping of iCy3 signals. Green asterisks represent iCy3 fluorophores and red circles represent MGME1 nucleases. **B**. The plots of population change of one-step and two-step NISP. **C.** Histograms of T_2 of MGME1 in degrading DNA substrate featuring a 3′ poly $(dT)_n$ overhang (n=20,40). Red lines indicate the nonlinear polynomial plus Gaussian fitting for T_2 distribution (in the presence of MGME1). These determined T_2 values are listed in Table 2

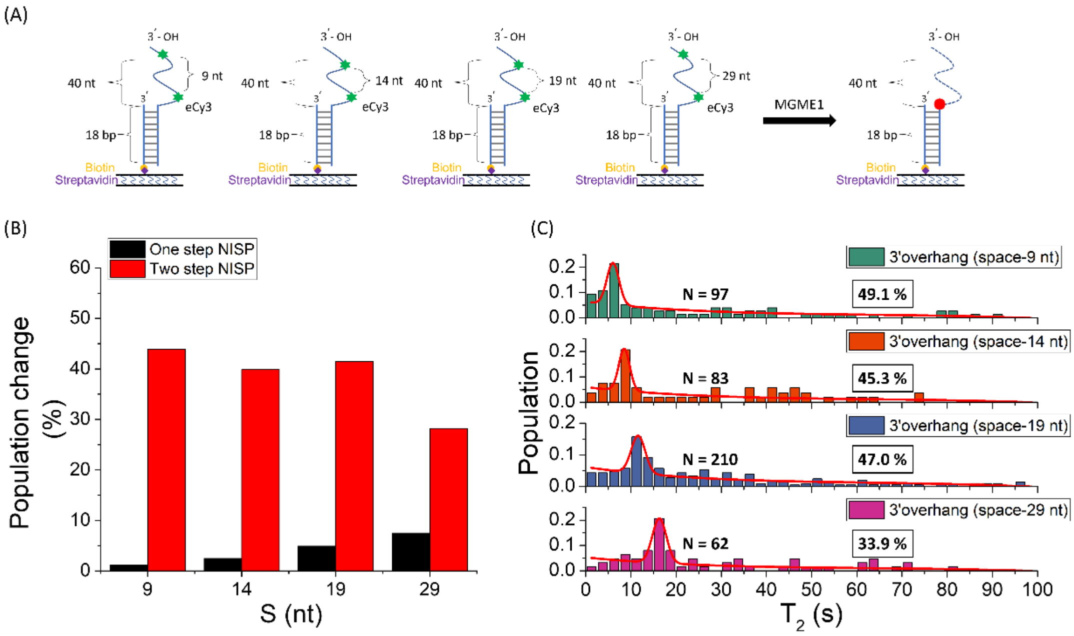

Supplementary Figure S13. *Length dependence of MGME1-mediated ssDNA degradation from 3' to 5' direction on di-eCy3 labeled DNA substrates.* **A**. Schematic illustrating of NISP for investigating ssDNA degradation by exonuclease, with a DNA substrate featuring a 3′ poly $(dT)_{40}$ overhang anchored on a PEGylating slide. The numbers of scissile phosphodiester bonds between two eCy3 fluorophores are 9, 14, 19, and 29 nt. After injection of exonuclease, ssDNA degradation can be detected by the stepwise photodropping of eCy3 signals. Green asterisks represent eCy3 fluorophores and red circles represent MGME1 nucleases. **B**. The plots of population change of one-step and two-step NISP. **C.** Histograms of T_2 of MGME1 in degrading DNA substrate featuring a 3′ poly $(dT)_{40}$ overhang. Red lines indicate the nonlinear polynomial plus Gaussian fitting for T_2 distribution (in the presence of MGME1). These determined T_2 values are listed in Table 2.

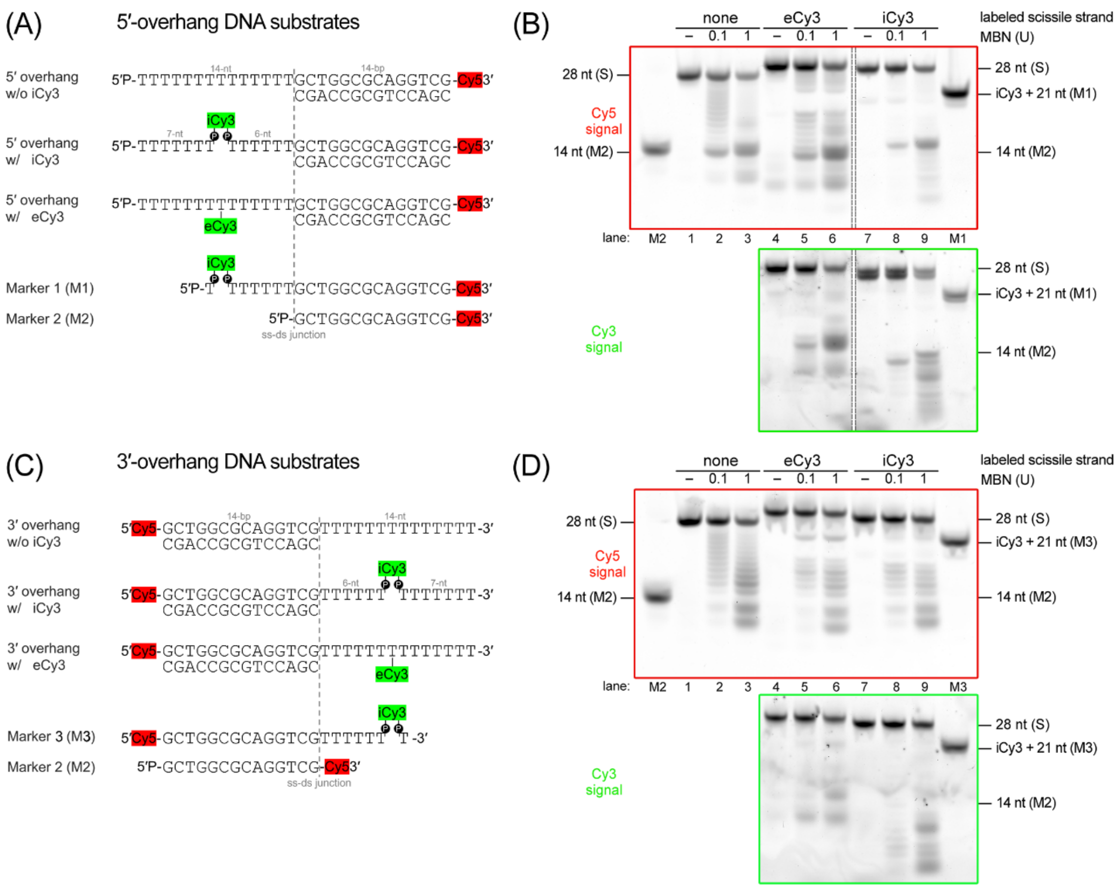

Supplementary Figure S14. *In-vitro nuclease assay of MBN in degrading Cy3-labeled overhang DNA.* The design of the substrate is shown in panels **A** and **C**. **B and D.** MBN in degrading the 14-nt 5′-overhang (**B**) and 3′-overhang (**D**) DNA. The unit (U) is defined according to the manufacturer (New England Biolabs). Each reaction (10 µL) contains 100 nM DNA substrate which was incubated with indicated units of MBN at 30°C for 5 minutes. After stopping the reaction with 0.1% SDS, the Cy5-labeled scissile strand was displaced from the complementary strand and separated with a 20% TBE acrylamide gel containing 7 M urea. Please refer to the Supplementary text for more description of the cleavage pattern. S, substrate.

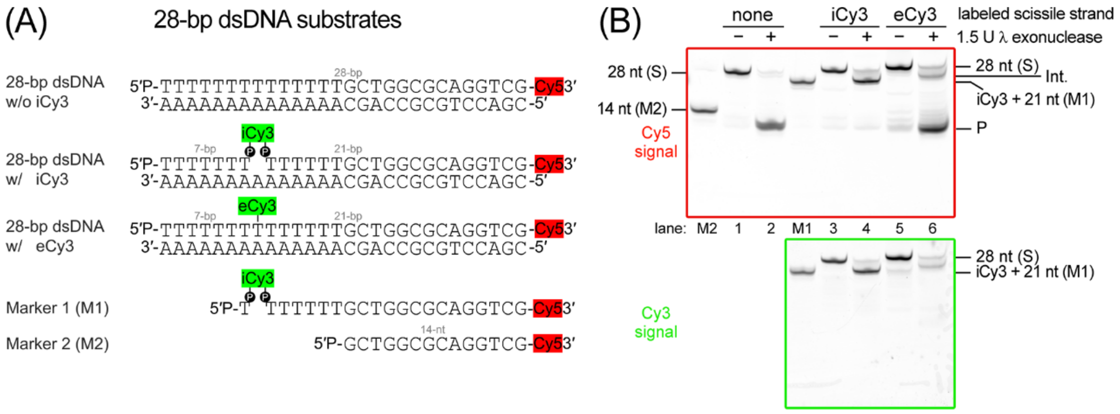

Supplementary Figure S15. *In-vitro nuclease assay of λ exonuclease in degrading Cy3-labeled overhang DNA.* (**A**) The design of the 28-bp dsDNA substrates. (**B**) λ exonuclease in degrading the 28-bp dsDNA substrates. Each reaction (10 μL) contains 100 nM DNA substrate which was incubated with 1.5 U λ exonuclease (New England Biolabs) at 37°C for one minute. After stopping the reaction, the Cy5-labeled scissile strand was displaced from the complementary strand and separated with a 20% TBE acrylamide gel containing 7 M urea. Please refer to the Supplementary text for more description of the cleavage pattern. S, substrate; P, products; Int., Cy3-induced cleavage intermediates.

Supplementary Table S1 The experimental DNA sequences

DNA substrate	Primer sequence	Experiment
5'→3'<br>di-iCy3-8nt[1]	5′-Phos-T***(iCy3)T***TTTTTTTT***(iCy3)T***TTTTTTTTGGC<br>G ACGGCAGCGAGGC<br>5′-Bio- GCCTC GCTGC CGTCG CCA	MGME1
5'→3'<br>di-iCy3-13nt[1]	5′-Phos-TTTTTTTTTTTTTTT***(iCy3)T***TTTTTTTTTTT<br>TT***(iCy3)T***TTTTTTTTGGCGACGGCAGCGAGGC<br>5-Bio- GCCTC GCTGC CGTCG CCA	MGME1
5'→3'<br>di-iCy3-18nt[1]	5′-Phos-TTTTTTTTTT***(iCy3)T***TTTTTTTTTTTTTTTT<br>TT***(iCy3)T***TTTTTTTTTGGCGACGGCAGCGAGGC<br>5′-Bio- GCCTC GCTGC CGTCG CCA	MGME1
5'→3'<br>di-iCy3-28nt[1]	5′-Phos-T***(iCy3)T***TTTTTTTTTTTTTTTTTTTTTTTTT<br>TTT***(iCy3)T***TTTTTTTTGGCGACGGCAGCGAGGC<br>5′-Bio- GCCTC GCTGC CGTCG CCA	MGME1
5'→3'<br>di-eCy3-9nt[1]	5′-Phos-TTTTTTTTTTTTTTTTTTTTT***T(eCy3)***TTTTTT<br>TTT***T(eCy3)***TTTTTTTTTGGCGACGGCAGCGAGG<br>C<br>5′-Bio- GCCTC GCTGC CGTCG CCA	MGME1
5'→3'<br>di-eCy3-14nt[1]	5′-Phos-TTTTTTTTTTTTTTTT***T(eCy3)***TTTTTTTTTTT<br>TTT***T(eCy3)***TTTTTTTTTGGCGACGGCAGCGAGG<br>C<br>5-Bio- GCCTC GCTGC CGTCG CCA	MGME1
5'→3'<br>di-eCy3-19nt[1]	5′-Phos-TTTTTTTTT***T(eCy3)***TTTTTTTTTTTTTTTTT<br>TT***T(eCy3)***TTTTTTTTTGGCGACGGCAGCGAGGC<br><br>5′-Bio- GCCTC GCTGC CGTCG CCA	MGME1<br>&<br>MBN
5'→3'<br>di-eCy3-29nt[1]	5′-Phos-***T(eCy3)***TTTTTTTTTTTTTTTTTTTTTTTT<br>TTTTT***T(eCy3)***TTTTTTTTTGGCGACGGCAGCGA<br>GGC<br>5′-Bio- GCCTC GCTGC CGTCG CCA	MGME1
3'→5'<br>di-iCy3-8nt[1]	5′-TGGCGACGGCAGCGAGGCTTTTTTTT***(iCy3)T***T<br>TTTTTTT***(iCy3)T***<br>5′- GCCTC GCTGC CGTCG CCA-Biotin	MGME1
3'→5'<br>di-iCy3-13nt[1]	5′-TGGCGACGGCAGCGAGGCTTTTTTTTT***(iCy3)T***<br>TTTTTTTTTTTTT***(iCy3)T***T TTTTTTTTTTTT<br>5′- GCCTC GCTGC CGTCG CCA-Biotin	MGME1

3′-to-5′<br>di-iCy3-18nt[1]	5′-TGGCGACGGCAGCGAGGCTTTTTTTTT***(iCy3)T***<br>TTTTTTTTTTTTTTTTTT***(iCy3)T***TTTTTTTTT<br>5′- GCCTC GCTGC CGTCG CCA-Biotin	MGME1
3’→5’<br>di-iCy3-28nt[1]	5′-TGGCGACGGCAGCGAGGCTTTTTTTT***(iCy3)T***T<br>TTTTTTTTTTTTTTTTTTTTTTTTTTT***(iCy3)T***<br>5′- GCCTC GCTGC CGTCG CCA-Biotin	MGME1
3’→5’<br>di-eCy3-9nt[1]	5′-TGGCGACGGCAGCGAGGCTTTTTTT TT***T(eCy3)***<br>TTTTTTTTT***T(eCy3)***TTTTTTTTTTTT TTTTTTTT<br>5′- GCCTC GCTGC CGTCG CCA-Biotin	MGME1
3’→5’<br>di-eCy3-14nt[1]	5′-TGGCGACGGCAGCGAGGCTTTTTTT<br>TT***T(eCy3)***TTTTTTTTTTTTTT***T(eCy3)***TTTTTTT<br>TTTTTTTT<br>5′- GCCTC GCTGC CGTCG CCA-Biotin	MGME1
3’→5’<br>di-eCy3-19nt[1]	5′-TGGCGACGGCAGCGAGGCTTTTTTTTT***T(eCy3)***<br>TTTTTTTTTTTTTTTTTTT***T(eCy3)***TTTTTTTTTT<br>5′- GCCTC GCTGC CGTCG CCA-Biotin	MGME1
3’→5’<br>di-eCy3-29nt[1]	5′-TGGCGACGGCAGCGAGGCTTTTTTT TT***T(eCy3)***<br>TTTTTTTTTTTTTTTTTTTTTTTTTTT TT***T(eCy3)***<br>5′- GCCTC GCTGC CGTCG CCA-Biotin	MGME1
5’→3’<br>di-iCy3-18nt<br>dsDNA[1]	5′-PhosTTTTTTTTTT***(iCy3)T***TTTTTTTTTTTTTTTTT<br>T***(iCy3)T***TTTTTTTTTGGCGACGGCAGCGAGGC<br>5′-Bio-GCCTCGCTGCCGTCGCCAAAAAAAAAAA<br>AAAAA AAAAAAAAAAAAAAAAAAAAAAAAA	MGME1
5’→3’<br>di-eCy3-9nt<br>dsDNA[1]	5′-PhosTTTTTTTTTTTTTTTTTTTT***T(eCy3)***TTTT<br>TTTTT***T(eCy3)***TTTTTTTTTTGGCG ACGGC AGCGA<br>GGC<br>5′-Bio-GCCTCGCTGCCGTCGCCAAAAAAAAAAA<br>AAAAA AAAAAAAAAAAAAAAAAAAAAAAAA	λ-<br>exonuclease
5’→3’<br>di-eCy3-14nt<br>dsDNA[1]	5′-PhosTTTTTTTTTTTTTTT***T(eCy3)***TTTTTTTTT<br>TTTTT***T(eCy3)***TTTTTTTTTTGGCG ACGGC AGCGA<br>GGC-<br>5′-Bio-GCCTCGCTGCCGTCGCCAAAAAAAAAAA<br>AAAAA AAAAAAAAAAAAAAAAAAAAAAAAA	λ-<br>exonuclease
5’→3’<br>di-eCy3-19nt<br>dsDNA[1]	5′-<br>PhosTTTTTTTTT***T(eCy3)***TTTTTTTTTTTTTTTTTT<br>T***T(eCy3)***TTTTTTTTTTGGCGACGGCAGCGAGGC<br>5′-Bio-GCCTCGCTGCCGTCGCCAAAAAAAAAAA<br>AAAAA AAAAAAAAAAAAAAAAAAAAAAAAA	λ-<br>exonuclease
FRET-dT_9<br>dsDNA	5′-Phos-TTTTTTTTTTTTTTTTTTTTTTTTTTTTT<br>***(iCy5)T***TTTTTTTTTGGCGACGGCAGCGAGGC<br>5′-Bio-GCCTCGCTGCCGTCGCC***(iCy3)A***AAAAAA<br>AAAAAAAAAAAAAAAAAAAAAAAAAAAAAAA<br>A	MGME1
FRET-19bp<br>dsDNA	5′-Phos-TGAGAATCGCCATATTTAACAGCCTC<br>GCTGCCGTCGCCA-Biotin<br>5′-TGGCGACGGCAGCGAGGC***T(eCy5)***GTT<br>AAATATGGCGATTCTC***A-Cy3***	λ-<br>exonuclease

20-nt DNA	5′-Phos-GCTTAACGCTGACTCGCTAC-FAM	*In vitro* nuclease assay (Supplementary Figure S6C)
7-nt 5′-overhang DNA	5′-Phos-GTCTAACGCTGACTCGCTACGTACC-FAM<br>5′-GGTACGTAGCGAGTCAGC	
25-nt competitive DNA	5′-GTCTAACGCTGACTCGCTACGTACC	
5′-overhang DNA	5′-Phos-TTTTTTTTTTTTTTTTTTTTTTTTTTTTTT<br>TTTTTTTTTT TGGCGACGGCAGCGAGG***C-Cy5***<br>5′-GCCTCGCTGCCGTCGCCA	*In vitro* nuclease assay (Supplementary Figure S11)
3′-overhang DNA	5′-FAM-TGGCGACGGCAGCGAGGCTTTTTTTTT<br>TTT TTTTTTTTTTTTTTTTTTTTTTTTTTTT<br>5′-GCCTCGCTGCCGTCGCCA	
18-nt competitive DNA	5′-TGGCGACGGCAGCGAGGC	
18-nt marker	5′-TGGCGACGGCAGCGAGG***C-Cy5***	
14-nt 5′-overhang	5′-Phos-TTTTTTTTTTTTTTGCTGGCGCAGGTC***G-<br>Cy5***<br>5′-CGACCGCGTCCAGC	*In vitro* nuclease assay (Supplementary Figures S2 and S14)
iCy3-labeled, 14-nt 5′-overhang DNA	5′-Phos-<br>TTTTTTT***(iCy3)T***TTTTTGCTGGCGCAGGTC***G-Cy5***<br>5′-CGACCGCGTCCAGC	
eCy3-labeled, 14-nt 5′-overhang DNA	5′-Phos-<br>TTTTTTT***T(eCy3)***TTTTTTGCTGGCGCAGGTC***G-<br>Cy5***<br>5′-CGACCGCGTCCAGC	
28-nt 5′ competitive DNA	5′-TTTTTTTTTTTTTTGCTGGCGCAGGTCG	
14-nt 3′-overhang	5′-Cy5-GCTGGCGCAGGTCGTTTTTTTTTTTTTT<br>5′-CGACCGCGTCCAGC	
iCy3-labeled, 14-nt 3′-overhang DNA	5′-Cy5-GCTGGCGCAGGTCGTTTTTT***(iCy3)T***TTT<br>TTT<br>5′-CGACCGCGTCCAGC	
eCy3-labeled, 14-nt 3′-overhang DNA	5′-Cy5-GCTGGCGCAGGTCGTTTTTT***T(eCy3)***TTT<br>TTTT<br>5′-CGACCGCGTCCAGC	

28-nt 3′ competitive DNA	5′-GCTGGCGCAGGTCGTTTTTTTTTTTTTT	
Marker 1	5′-Cy5-GCTGGCGCAGGTCGTTTTTT***(iCy3)T***	*In vitro* nuclease assay (Supplementary Figures S2, S14 and S15)
Marker 2	5′-Phos-GCTGGCGCAGGTC***G-Cy5***	
Marker 3	5′-Cy5-GCTGGCGCAGGTCGTTTTTT***(iCy3)T***	
28-bp dsDNA	5′-Phos-TTTTTTTTTTTTTTGCTGGCGCAGGTC***G-Cy5***<br>5′-CGACCGCGTCCAGCAAAAAAAAAAAAAA	*In vitro* nuclease assay (Supplementary Figure S15)
iCy3-labeled, 28-bp dsDNA	5′-Phos-TTTTTTT***(iCy3)T***TTTTTGCTGGCGCAGGTC***G-Cy5***<br>5′-CGACCGCGTCCAGCAAAAAAAAAAAAAA	
eCy3-labeled, 28-bp dsDNA	5′-Phos-TTTTTTT***T(eCy3)***TTTTTGCTGGCGCAGGTC***G-Cy5***<br>5′-CGACCGCGTCCAGCAAAAAAAAAAAAAA	

[1]: This value represents the number of nucleotides with scissile phosphodiester bonds between the two Cy3 fluorophores on the DNA substrate.

*The texts in bold, italic and underlined represent the Cyanine fluorophore labeled nucleotides

Supplementary References

1. Yang, C., Wu, R.Q., Liu, H.H., Chen, Y.Q., Gao, Y.Q., Chen, X., Li, Y.Y., Ma, J.B., Li, J.X. and Gan, J.H. (2018) Structural insights into DNA degradation by human mitochondrial nuclease MGME1. *Nucleic Acids Research*, **46**, 11075-11088.
2. Mao, E.Y.C., Yen, H.Y. and Wu, C.C. (2024) Structural basis of how MGME1 processes DNA 5´ ends to maintain mitochondrial genome integrity. *Nucleic Acids Research*, **52**, 4067-4078.
3. Schneider, C.A., Rasband, W.S. and Eliceiri, K.W. (2012) NIH Image to ImageJ: 25 years of image analysis. *Nat Methods*, **9**, 671-675.
